# Synergistic epistasis among cancer drivers can rescue early tumors from the accumulation of deleterious passengers

**DOI:** 10.1371/journal.pcbi.1012081

**Published:** 2024-04-30

**Authors:** Carla Alejandre, Jorge Calle-Espinosa, Jaime Iranzo

**Affiliations:** 1 Centro de Astrobiología (CAB) CSIC-INTA, Torrejón de Ardoz, Madrid, Spain; 2 Centro de Biotecnología y Genómica de Plantas, Universidad Politécnica de Madrid (UPM)—Instituto Nacional de Investigación y Tecnología Agraria y Alimentaria (INIA-CSIC), Madrid, Spain; 3 Institute for Biocomputation and Physics of Complex Systems (BIFI), University of Zaragoza, Zaragoza, Spain; University of California San Diego Division of Biological Sciences, UNITED STATES

## Abstract

Epistasis among driver mutations is pervasive and explains relevant features of cancer, such as differential therapy response and convergence towards well-characterized molecular subtypes. Furthermore, a growing body of evidence suggests that tumor development could be hampered by the accumulation of slightly deleterious passenger mutations. In this work, we combined empirical epistasis networks, computer simulations, and mathematical models to explore how synergistic interactions among driver mutations affect cancer progression under the burden of slightly deleterious passengers. We found that epistasis plays a crucial role in tumor development by promoting the transformation of precancerous clones into rapidly growing tumors through a process that is analogous to evolutionary rescue. The triggering of epistasis-driven rescue is strongly dependent on the intensity of epistasis and could be a key rate-limiting step in many tumors, contributing to their unpredictability. As a result, central genes in cancer epistasis networks appear as key intervention targets for cancer therapy.

## Author summary

During life, cell populations accumulate mutations that can turn them into tumors. Such mutations, called drivers, do not act alone: they often potentiate each other in a synergistic way. Some recent works suggest that the fate of nascent tumors could depend on a tug-of-war between tumor-promoting drivers and slightly deleterious “passenger” mutations that occur at much faster rates and hinder tumor progression. We studied how synergy among drivers affects the balance between drivers and passengers and, with that, the probability that nascent tumors progress to cancer. Our results, based on computer simulations, mathematical modeling, and empirical data, show that synergistic interactions among drivers overcome the effect of passenger mutations, substantially increasing the risk of cancer and reducing the predictive power of mutation profiling for early detection. On the positive side, characterization of synergies among cancer genes could help us identify better targets for cancer therapy in the future.

## Introduction

Tumor development is an evolutionary process driven by somatic mutations and selection in the context of rapidly adapting cell populations [1–4]. In that process, tumor cells acquire mutations (driver mutations or just *drivers*) that confer a growth advantage and facilitate tumor proliferation, spreading, and colonization of new niches. Drivers coexist in the genome of cancer cells with a much larger number of passenger (non-driver) mutations (or just *passengers*) [5–7]. The role of passengers in cancer evolution has often been neglected, assuming that they have no or little impact on the cancer phenotype. However, a growing body of evidence suggests that the accumulation of slightly deleterious passengers could play important roles in adaptive processes [8–10] and hinder the proliferation of cancer cells [11–13].

Recent sequencing of normal human tissues has revealed that somatic mutations are pervasive among non-neoplastic cells [14–23]. These mutations include potential drivers in well-known cancer genes, such as *TP53* or *KRAS*, and have been reported in a variety of tissues such as skin, esophagus, and bladder. The presence of cancer drivers in apparently healthy tissues implies that drivers are necessary, but often not sufficient, for neoplastic transformation. Among the additional necessary factors, recent studies have pointed at chromosome instability and high mutation burden, the latter accounting for a higher number of drivers than what is commonly found in normal cells [19,24]. Sustained proliferation of emergent micro-tumors could also be suppressed by clonal interference, that is, by competition with other mutant clones, as observed in animal models of epithelial cancer [23]. More generally, the fates of mutant clones depend on the interactions between their driver mutations, the genetic background in which they appear, and the tissue microenvironment. Such gene-gene and gene-environment interactions, most of which remain poorly understood, could explain why cell populations with multiple driver alterations do not develop into cancer.

Epistasis, that is, the variation of the fitness effect of a mutation depending on the genetic background, is a major consequence of gene-gene interactions. Synergistic and antagonistic epistasis among drivers has been widely reported based on patterns of co-occurrence and mutual exclusivity [25–27] and laboratory assays with tumor cell lines [28]. Two classical examples are the synergistic interaction between activating mutations in *MYC* and amplification of *RAS* [29] and the mutually exclusive relationship between alterations in *KRAS* and *BRAF* across many cancer types [30]. More recently, systematic approaches have identified large epistasis networks encompassing dozens of genes and hundreds of pairwise interactions in different cancer types [31,32]. Epistasis networks are highly cancer-type specific and display a large variety of structural features, such as star-like motifs centered in a master cancer gene and dense clique-like motifs in which all genes interact with each other. These motifs sometimes include synergistic mutations in genes from the same pathway [33,34] that challenge the traditional notion of within-pathway redundancy [35]. Although most efforts have focused on finding synthetic-lethal partners with potential therapeutic application [36–40], the study of synergistic (or positive) epistasis, whereby the presence of a driver enhances the effect of another driver, could shed light on relevant aspects of tumor development, such as convergence towards well-differentiated subtypes [32] and differential response to therapy [31,41].

In this work, we combine empirical epistasis networks, mathematical models, and computer simulations to study how positive epistasis affects the probability of developing rapidly-growing tumors in 10 different cancer subtypes. We describe and formally characterize a phenomenon of epistasis-driven evolutionary rescue that allows for cancer progression in conditions in which the acquisition of drivers alone would be insufficient to compensate for the mutational burden imposed by passenger mutations [42,43]. For two simple network structures, star and clique, we provide approximate analytical expressions for the probability of rescue and the time at which it occurs, and study the impact of mutation rate, epistasis strength, and network topology on triggering tumor rescue. By facilitating the evolutionary rescue of tumors, positive epistasis increases the unpredictability of cancer evolution. Finally, we show that epistasis can modify the prevalence of driver alterations in cell populations that do not progress into cancer.

## Results

### Model of cancer evolution

**Fig 1:**
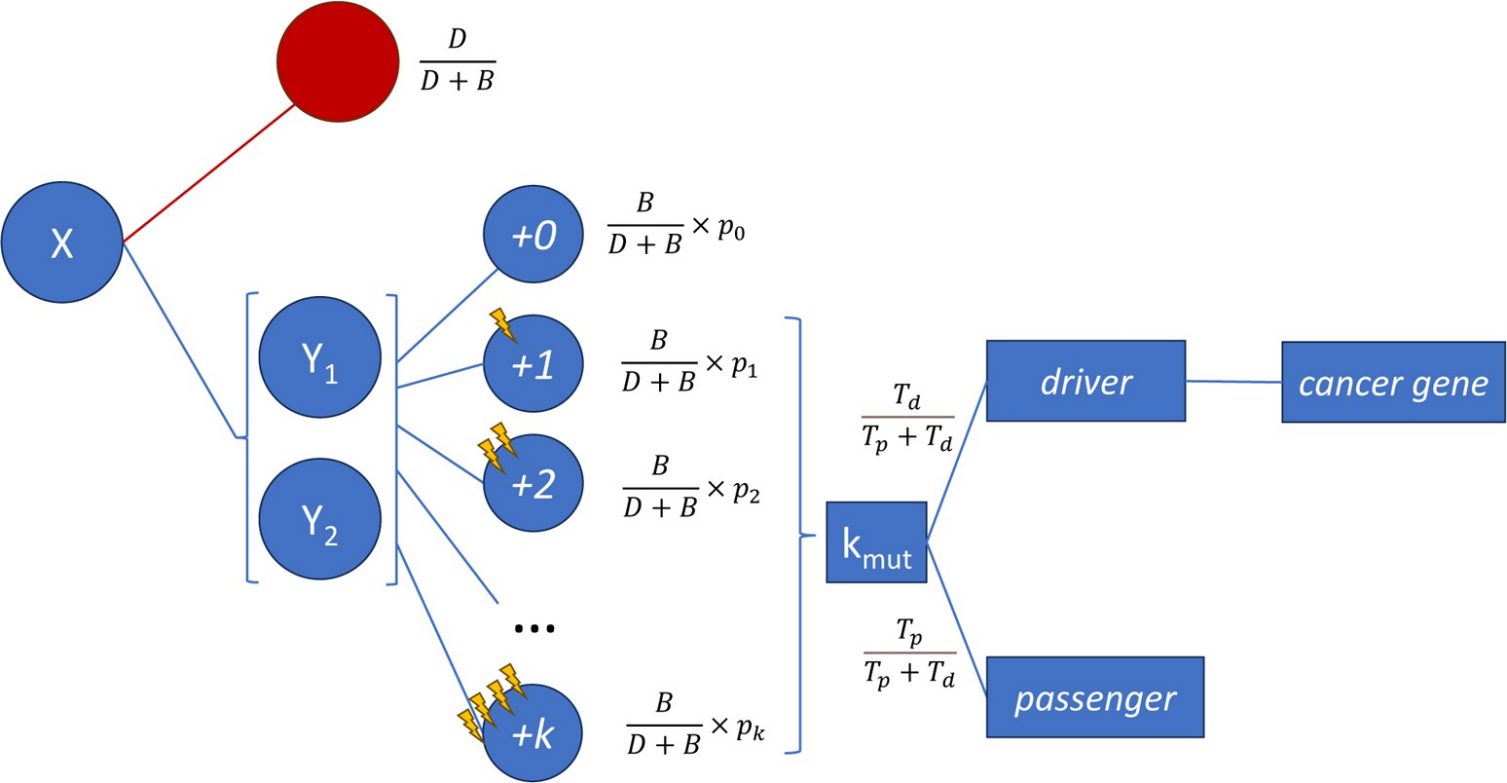
Diagram of the generalized birth-and-death model of cancer evolution. At each generation, each cell (X) can either die (red) or divide (blue) and produce two daughter cells (Y_1_ and Y_2_) with probabilities that depend on the death (D) and birth (B) rates of the system. For each daughter cell, the probability that it acquires +0, +1, +2, …, +*k* mutations (*p*_*k*_) is given by a Poisson distribution with mean *μ*(*T*_*d*_+*T*_*p*_). The new mutations (k_mut_) are catalogued as drivers with probability $\frac{T_{d}}{T_{p}+T_{d}}$ or passengers with probability $\frac{T_{p}}{T_{p}+T_{d}}$. Driver mutations are randomly assigned to a cancer gene, that may or may not be involved in the epistasis network. The definitions of the parameters and the expressions of the birth and death rates are provided in the main text and Table 1.

We simulated the evolution of a cancer cell population as a generalized birth-and-death process that accounts for slightly deleterious passengers and synergistic epistasis among drivers. Simulations start from a precancerous lesion that has reached a size (*N*_0_) equal to the carrying capacity of its local microenvironment. Such carrying capacity can be determined, for example, by nutrient availability, physical constraints, or the interaction with competing clones. The simulations then proceed in discrete generations during which the whole cell population is synchronously updated (Fig 1). At each generation, each cell divides or dies with probabilities $\frac{B}{B+D}$ and $\frac{D}{B+D}$, respectively. Following a popular model of cancer dynamics originally introduced by McFarland et al. [42,43], we assumed that the death rate *D* is density-dependent ($D=\frac{N}{N_{0}}$) and the division rate *B* is fitness-dependent. The fitness is determined by the mutations carried by each cell, as described below. We considered two types of mutations: (i) slightly deleterious passenger mutations that reduce fitness and (ii) driver mutations that enable cells to grow beyond the limitations imposed by the initial carrying capacity. Thus, the fitness of a cell with *n*_*d*_ drivers and *n*_*p*_ passengers is equal to $B=\frac{{\left(1+s_{d}\right)}^{n_{d}}}{{\left(1+s_{p}\right)}^{n_{p}}}$, where *s*_*d*_ and *s*_*p*_ are the relative fitness effects associated with drivers and passengers in the absence of epistasis. We assumed constant *s*_*d*_ and *s*_*p*_ for simplicity, although previous works have shown that the phenomenology does not qualitatively change if the fitness effects are random variables drawn from log-normal, exponential, or gamma distributions, at least in the absence of epistasis [42]. In the absence of mutations, the equilibrium population size is reached when *D* = *B* = 1, which justifies the interpretation of *N*_0_ as the basal carrying capacity.

We assumed that mutations occur when cells divide and can affect any of the daughter cells. This choice is motivated by the empirical findings that the probability of developing tumors in different tissues is proportional to the number of stem cell divisions [44] and that the frequency of somatic mutations is better predicted by the number of cell divisions than by absolute time [45]. The number of new mutations per cell division is taken from a Poisson distribution with mean *μT*_*d*_ for driver mutations and *μT*_*p*_ for passenger mutations, where *μ* is the mutation rate, *T*_*d*_ is the number of potential driver sites in the genome, and *T*_*p*_ is the number of potential passenger sites. In general, the growth-promoting effect of drivers depends on the mutational background and the epistasis profile. Accordingly, to model synergistic interactions among drivers, we considered an underlying network of gene-level epistasis and explicitly assigned each driver mutation to one out of *G* cancer genes. We then multiplied the fitness effect of each driver by a factor *ϵ* if any of the neighbor genes in the epistasis network also harbors driver mutations. As a result, the fitness becomes $B=\frac{{\left(1+s_{d}\right)}^{n_{r}}{\left(1+{\epsilon s}_{d}\right)}^{n_{e}}}{{\left(1+s_{p}\right)}^{n_{p}}}$, where *n*_*r*_ is the number of “regular” drivers and *n*_*e*_ is the number of drivers involved in synergistic fitness interactions (enhanced drivers). To account for the fact that redundant driver mutations hitting the same cancer gene do not generally increase fitness, we only allowed one driver per cancer gene, multiplying the driver target size *T*_*d*_ by a factor $\frac{G-1}{G}$ and reducing *G* in one unit every time that a new driver is assigned (note that, as a result, *T*_*d*_ and *G* vary across cells and along the simulation).

The relevance of epistasis is quantified by two parameters: its maximum spread (that is, the fraction *f*_*e*_ of the *G* original cancer genes that participate in the epistasis network) and its magnitude (that is, the factor *ϵ* by which the fitness effect of a driver is amplified). Note that the limit *f*_*e*_ = 1, *ϵ*→∞ (with *s*_*d*_ properly rescaled to keep the product *ϵs*_*d*_ constant) is formally equivalent to Knudson’s two-hit hypothesis [46], in the sense that the first driver event is effectively neutral, while the second can initiate cancer. Despite its relative simplicity, this model reproduces some key features of cancer evolutionary dynamics, such as gompertzian growth, emergence of co-occurrence and mutual exclusivity patterns among drivers, and spontaneous differentiation into mutational subtypes [31,32].

We ran the simulations for 30,000 cell generations, which is higher than the actual number of cell generations in a lifetime. (For example, assuming an 80-year lifespan and one division every 2–3 days for gastrointestinal stem cells [47], the maximum number of cell generations would be around 10,000–15,000.) By extending the simulations to longer times, we ensured that the results for the probability of developing cancer could be interpreted as conservative upper bounds. We discuss how this choice affects qualitative trends in the section about the timing of tumor rescue. Other default parameter values are listed in Table 1. We classified the outcome of each simulation as tumor progression if the final size of the cell population had doubled the original *N*_0_. For each parameter combination, we ran 1000 independent simulations to estimate the probability of tumor progression. Technical details concerning the implementation of the simulations are discussed in the Methods section. We provide the code for the simulations as a supplementary file (S1 File).

**Table 1:** Default parameter values used in the simulations.

Parameter	Value	Description
***μ***	10^−9^−10^−8^	Mutation rate per site
***s***_***d***_	0.01−0.1	Fitness effect of drivers
***s***_***p***_	10^−3^	Fitness effect (cost) of passengers
***ϵ***	0−10	Epistasis factor
***f***_***e***_	0−1	Fraction of cancer genes in the epistasis network
***T***_***d***_	100	Mutational target size for drivers
***T***_***p***_	5×10^6^	Mutational target size for passengers
***N***_**0**_	10^3^	Carrying capacity
***G***	70	Number of cancer genes

### Synergistic epistasis can induce evolutionary rescue of receding tumors

**Fig 2:**
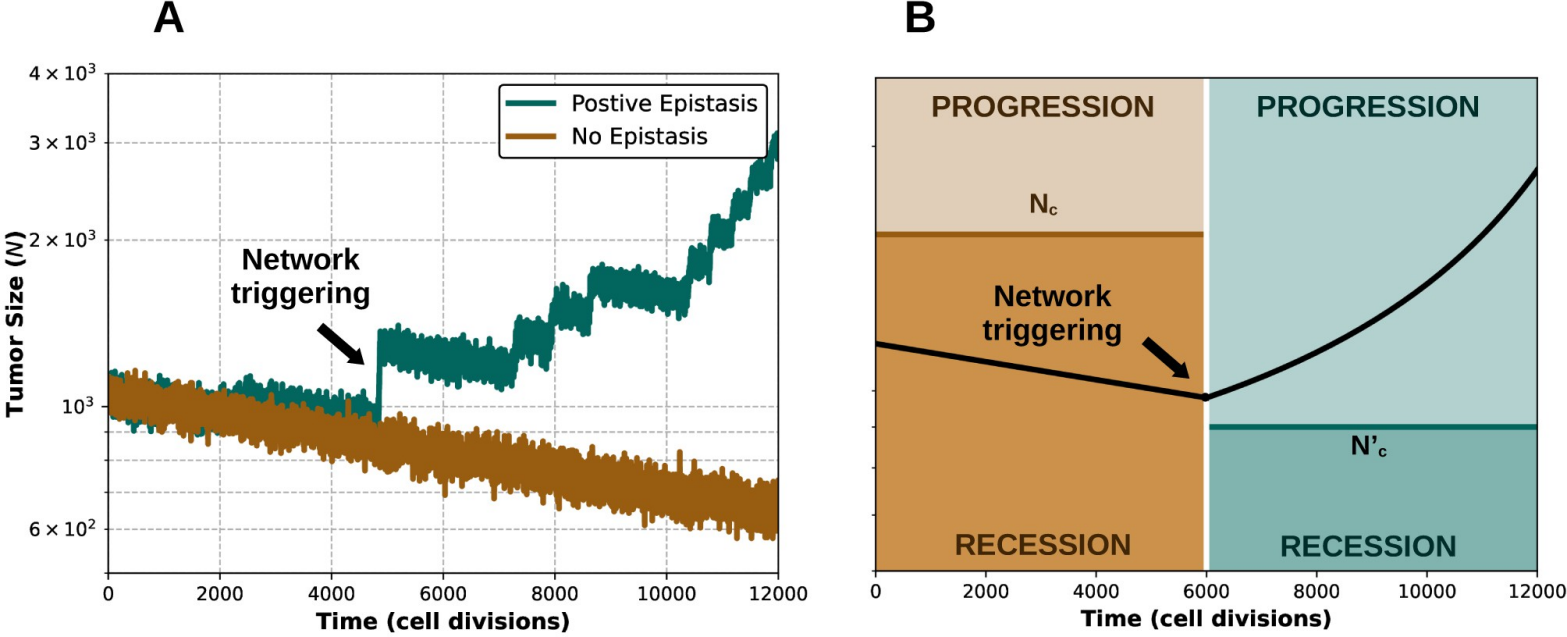
Synergistic epistasis can induce evolutionary rescue of receding tumors. (A) Representative trajectory of a precancerous lesion subject to synergistic epistasis among drivers (green), superimposed to a representative trajectory without epistasis (brown). Parameter values: *μ* = 10^−8^, *s*_*d*_ = 0.07, *ϵ* = 2, *f*_*e*_ = 0.5, *G* = 70, *N*_0_ = 10^3^. (B) Schematic of the dynamics of an initial receding tumor that is rescued by a trigger driver that appears after approximately 6000 cell divisions. *N*_*c*_ and $N_{c}^{\prime }$ represent the critical population sizes before and after the trigger event, respectively. For the rescue to occur, the trigger driver must appear before the population size drops below $N_{c}^{\prime }$.

Previous works that used a similar mathematical model to investigate the effect of deleterious passengers on cancer progression found that the fate of the precancerous lesion depends on a tug-of-war between drivers and passengers [42,43]. This tug-of-war can be intuitively explained by comparing the probabilities of appearance and fixation of driver and passenger mutations in the cancer cell population (S1 Text) [42,43]. On the one side, the rate of appearance and fixation of beneficial drivers is approximately proportional to *s*_*d*_*T*_*d*_*N*, where *T*_*d*_ is the mutational target size associated with drivers. On the other side, slightly deleterious passengers accumulate in a nearly neutral way, at a rate proportional to the size of the passenger mutational target *T*_*p*_. Because the mutational target is much larger for passengers than drivers, if the initial lesion is small, the appearance and fixation of new drivers is often too slow to compensate for the much faster accumulation of passengers, hindering tumor progression. In contrast, in larger lesions, the supply of drivers can surpass the deleterious effect of passengers, leading to rapid tumor growth. In the absence of epistasis, these two regimes are separated by a critical population size (*N*_*c*_), such that the initial lesion will almost surely progress to cancer if *N*_0_>*N*_*c*_ and recede if *N*_0_<*N*_*c*_. However, our simulations show that if the model includes synergistic epistasis, lesions that are initially in the receding regime can sometimes transition into the cancer regime (Fig 2A). Such transitions are the consequence of driver mutations in key cancer genes that act as cancer facilitators by enhancing the fitness effects of other (often less prominent) drivers.

The change of regime, whereby a receding cell population turns into a rapidly growing tumor, constitutes a case of evolutionary rescue. Synergistic epistasis facilitates rescue because it amplifies the fitness effect of growth-promoting drivers, flipping the balance between these and deleterious passengers. From a formal perspective, the cause of the transition is a drop in the critical population size. Indeed, by approximating the dynamics of mutation accumulation as a deterministic process, it can be shown (section 1.2 in S1 Text) that the critical size is inversely proportional to the square of the fitness effect associated with driver mutations, that is $N_{c}∼{1/s}_{d}^{2}$. Therefore, if drivers in a key cancer gene increase the fitness effect of all other drivers by a factor *ϵ*, the critical size will decrease by a factor approximately equal to *ϵ*^2^. This change will be enough to rescue receding populations whose size lay above the new critical size (Fig 2B).

### Evolutionary rescue in real-world epistasis networks

**Fig 3:**
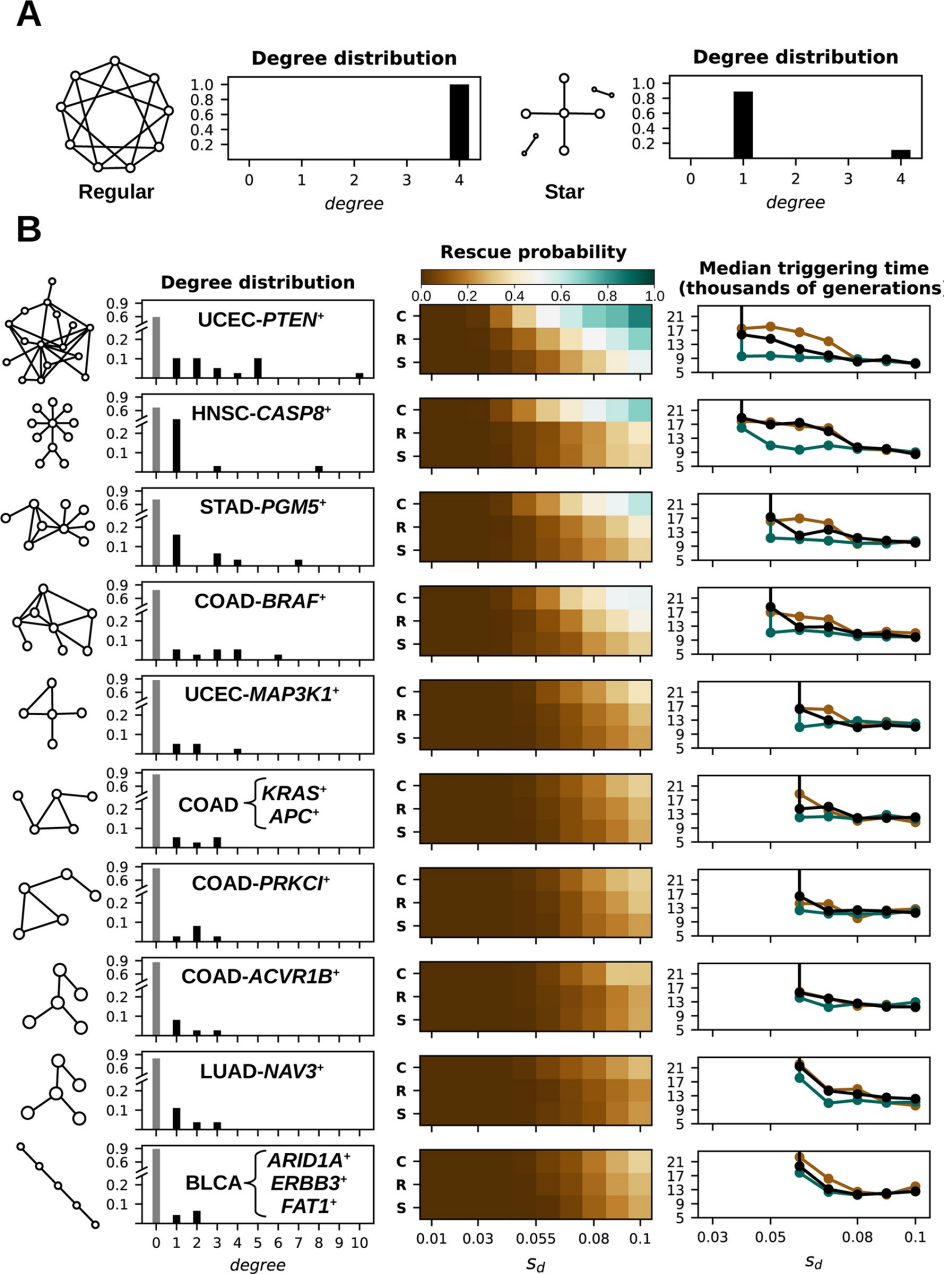
Tumor rescue in real-world epistasis networks. (A) Simplified representation of regular (left) and star-like (right) networks. The actual star and clique networks consist of up to 70 genes. (B, from left to right) Structure of positive epistasis networks for several representative cancer subtypes; their degree distributions; comparison of the probability of tumor progression among regular (C), representative cancer subtype (R), and star-like (S) networks; and median triggering time in regular (green), representative cancer subtype (black), and star-like (brown) networks. For each cancer subtype, the rescue probability and trigger time of the empirical epistasis network are compared with regular and star-like networks with the same hub connectivity (see Methods). The gray bars in the degree distribution correspond to the fraction of nodes that are not affected by epistasis. Networks for representative cancer subtypes are based on the modular structure of empirical epistasis networks (the identity of the most connected cancer gene is indicated in each case). The triggering time is determined by the occurrence of the first driver that makes $N_{c}^{\prime }<N_{0}$. Parameter values: *N*_0_ = 10^3^, *s*_*p*_ = 10^−3^, *T*_*p*_ = 5×10^6^, *ϵ* = 6, *μ* = 5×10^−9^, variable *T*_*d*_ according to the number of cancer genes associated with each cancer type.

In more realistic scenarios, the effect of epistasis on cancer progression depends on the structure of the epistasis network. From here on, we use the term *trigger driver* to denote those mutations that significantly amplify the fitness effects of many other drivers. Trigger drivers are associated with genes that act as hubs (that is, highly connected nodes) in the epistasis network. For example, in star-like networks (Fig 3A, right), mutations in the central gene act as triggers because they affect all other genes connected to it. In dense regular networks (Fig 3A, left), the first mutation in any gene of the network can act as the trigger, affecting most other genes. The structural heterogeneity of real-world epistasis networks (Fig 3B, left) allows for multiple triggers, not all equally powerful. The trigger event modifies the critical size required for tumor progression, from a pre-trigger size *N*_*c*_ to a post-trigger size $N_{c}^{\prime }$ (with $N_{c}^{\prime }<N_{c}$). These two critical sizes delimit the region in which evolutionary rescue is possible. More precisely, precancerous lesions with initial size between *N*_*c*_ and $N_{c}^{\prime }$ will initially decay but can be rescued and progress to tumors if trigger drivers appear and reach high prevalence early enough in the process. Because rescue is extremely rare once the population size has fallen below $N_{c}^{\prime }$, trigger drivers do not guarantee rescue. In turn, tumor rescue is a stochastic process that greatly depends on the temporal dynamics of trigger drivers.

Simulations with empirical epistasis networks and biologically reasonable values of other model parameters (Table 1) show that evolutionary rescue is feasible in multiple cancer types (Fig 3B, middle). Comparing among epistasis networks, the single property that most strongly affects the probability and timing of rescue is the connectivity of the hubs. Because of that, the dynamics of tumor rescue in real-world networks can be approximated by studying two extreme cases with the same hub connectivity, namely star-like and regular networks.

### Tumor rescue is facilitated by high mutation rates and strong epistasis

**Fig 4:**
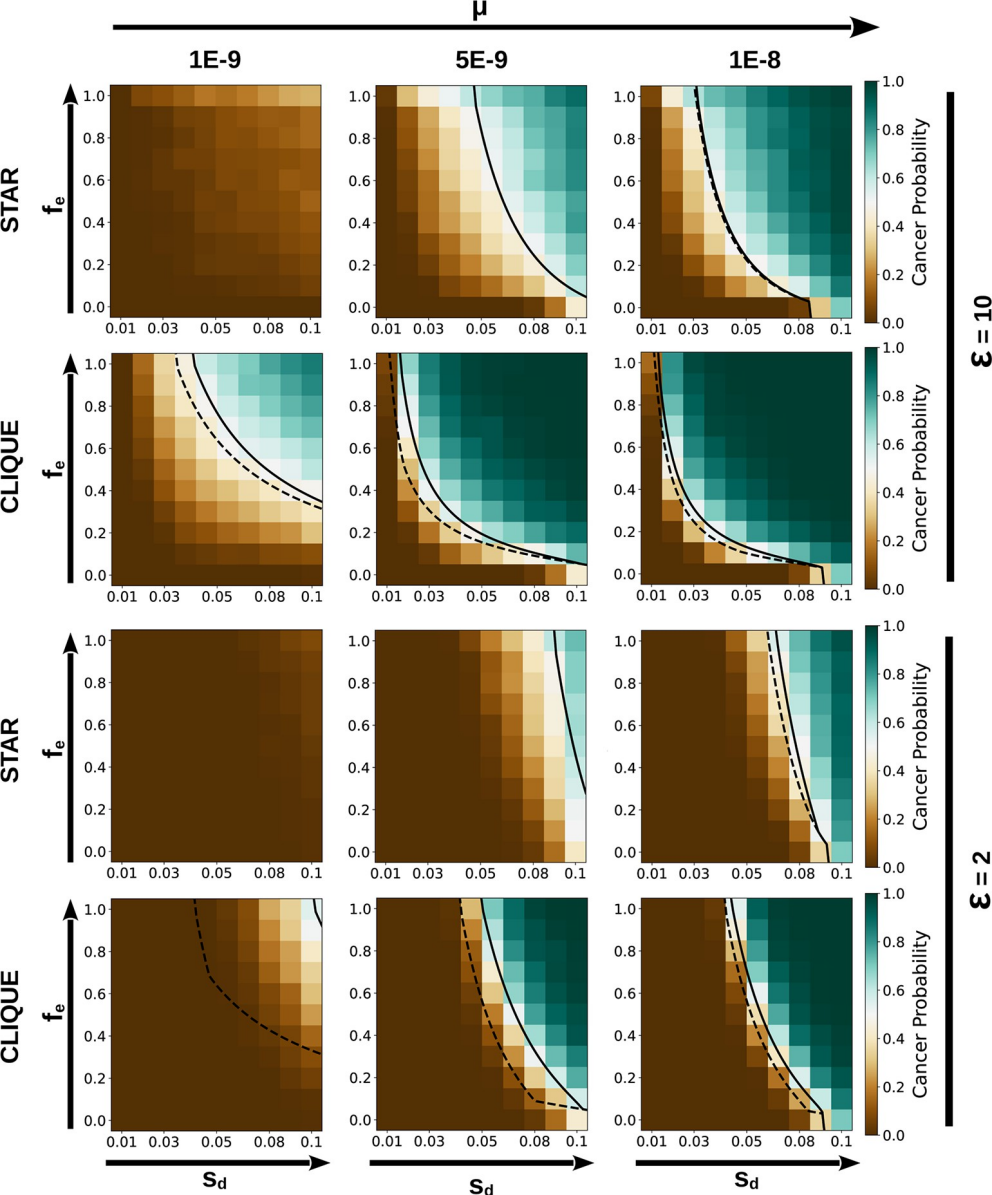
Tumor rescue is facilitated by high mutation rates and strong epistasis. Probability of tumor progression as a function of network structure, mutation rate (*μ*), driver fitness effect (*s*_*d*_), fraction of genes subject to epistasis (*f*_*e*_), and strength of epistasis (*ϵ*). The probabilities were calculated for 1000 independent trajectories and each trajectory was classified as “tumor progression” if the population doubled its size before 30,000 cell divisions. The solid and dashed lines correspond to the analytical curves for a probability of 0.5 when taking and not taking into account the upper time limit of 30,000 cell divisions, respectively. In the region between the two curves, tumor progression is technically possible, but too slow to become manifest during human lifespan. Parameter values: *N*_0_ = 10^3^, *s*_*p*_ = 10^−3^, *T*_*p*_ = 5×10^6^, *T*_*d*_ = 700, *G* = 70.

Star-like and regular networks represent two extremes in terms of clustering coefficient (1 in the regular network, 0 in the star) and degree heterogeneity (maximum in the star, minimum in the regular network). Moreover, they provide lower and upper bounds to the rescue probabilities and trigger times observed in real-world epistasis networks. Due to their simplicity, stars and cliques (a particular class of regular networks with full connectivity) are especially attractive to perform analytical and numerical calculations. Thus, to better understand the factors that determine evolutionary rescue in tumors, we focused on star- and clique-like networks and estimated their rescue probabilities by means of computer simulations and approximate analytical calculations. Both approaches are in excellent agreement (*R*^2^ = 0.987, S1 Fig) and show that the rescue probability increases with the mutation rate (*μ*), the fitness effect of driver mutations (*s*_*d*_), the strength of epistasis (*ϵ*), and the fraction of cancer genes subject to positive epistasis (*f*_*e*_) (Figs 4 and S2; the bottom row in each panel, *f*_*e*_ = 0, corresponds to the case without epistasis). Compared to the case without epistasis, synergistic interactions among driver mutations can notably increase the risk that precancerous lesions progress to cancer (white and blue regions in Fig 4), especially if epistasis is strong or the somatic mutation rate is high (*μ*≥5×10^−9^ per bp per replication cycle, which lies in lower the range of mutator phenotypes [48]). For the range of parameters explored in this study, positive epistasis is needed to explain cancer progression at low somatic mutation rates (10^−9^ per bp per replication cycle), while keeping driver fitness effects compatible with those reported in the literature [43,49,50]. The analytical solution of the model also allows identifying parameter regions where tumors can grow, but so slowly that they rarely double their initial size during a lifetime (Fig 4, region delimited by dashed and full lines). Such slow-growing tumors are more likely to appear under low mutation rates and weak (but widespread) epistasis (lower left panel in Fig 4).

For the range of biologically plausible parameter values explored in this work, the rescue probability in clique-like epistasis networks is always greater than in star-like networks (S2 Fig). The main reason for this difference is the size of the mutational target associated with trigger drivers, which are restricted to a single gene (the hub) in the star but can hit any gene in the clique.

### The timing of tumor rescue is critically affected by the structure of epistasis networks

**Fig 5:**
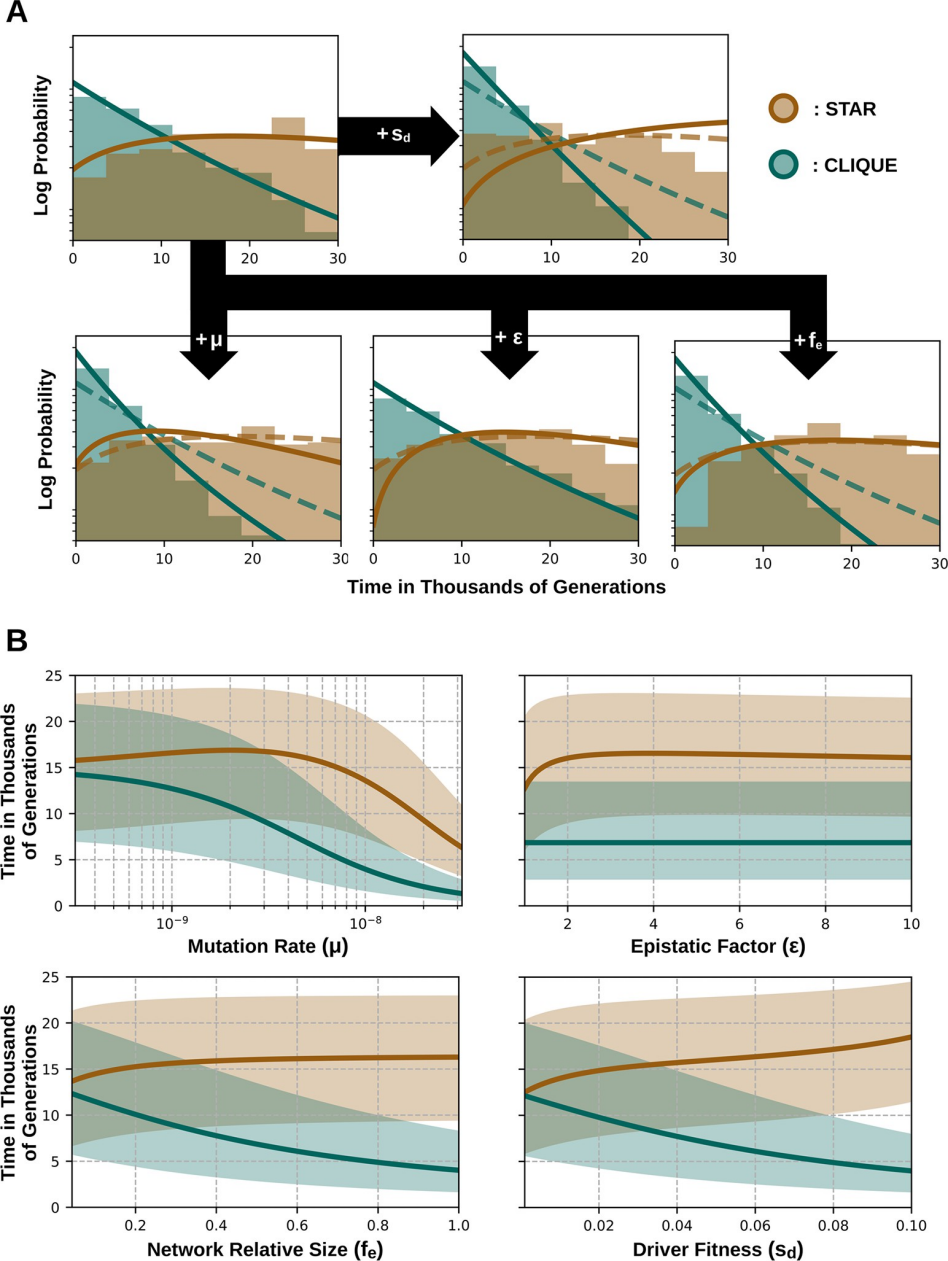
Timing of tumor rescue is critically affected by the structure of the epistasis network. (A) Distribution of tumor rescue times (understood as the time of appearance of a trigger driver in the most connected gene) in star- (brown) and clique-like (green) networks, and their variation with the mutation rate (*μ*), driver fitness effect (*s*_*d*_), fraction of genes subject to epistasis (*f*_*e*_), and strength of epistasis (*ϵ*). Histograms were obtained from 1000 independent trajectories; solid lines correspond to analytical expressions. To facilitate comparisons, all the distributions are normalized to the same total area. (B) Dependence of the median rescue times (solid lines) and their 25–75 percentiles (shaded areas) on the parameters of the model, based on analytical expressions. Parameter values: *μ* = 5×10^−9^, *s*_*d*_ = 0.05, *f*_*e*_ = 0.5, *ϵ* = 2, G = 70, rest of parameters as in Fig 2B.

The temporal dynamics of tumor rescue are governed by the stochastic appearance and subsequent fixation of the trigger driver in the receding cell population. Understanding these dynamics is important because they could affect the demographics and predictability of different cancer types. Simulations and analytical calculations show that the timing of tumor rescue greatly varies between clique- and star-like epistasis networks (Fig 5). In the clique, the distribution of rescue times has a maximum at time zero and then decays almost exponentially, such that $\log P(t)\propto -\mu s_{d}f_{e}t$ (section 1.4 in S1 Text). In contrast, rescue times in the star follow a flatter distribution with a less prominent maximum at intermediate times (Fig 5A). Such differences are related to the possibility that drivers in peripheral genes of the star reach fixation before the trigger driver occurs. More precisely, because the average number of drivers in peripheral genes increases with time and pre-existent peripheral drivers facilitate the fixation of the trigger driver, the rescue probability in the star also increases with time. The maximum at intermediate times results from a trade-off between the higher probability of fixation of the trigger driver at longer times and the decrease in the total population size (and therefore, in the probability that the trigger driver occurs) with time.

Rescue time distributions allow assessing how setting an upper limit for the number of cell generations affects the probability of developing cancer. In clique-like networks, if rescue occurs, it happens early (usually before 10,000 generations). In contrast, in star-like networks, rescue occurs later and spans a wider time window (around 10,000–20,000 generations). Because of that, tissue-specific variations within a range of 10,000–15,000 maximum cell generations (which is a realistic assumption for a human lifetime) will substantially affect the rescue probability of tumors with star-like epistasis networks, but not of those with clique-like networks.

The effect of different parameters on the median rescue time depends on the topology of the epistasis network (Fig 5B). Thus, in the clique, the median rescue time decreases with the mutation rate, the size of the epistasis network, and the fitness effect of drivers. In contrast, the median rescue time in the star remains approximately invariant with respect to the size of the epistasis network, slightly increases with the fitness effect of drivers, and only decreases at high mutation rates. In both types of networks, the median rescue time is independent of the strength of epistasis. Finally, for any fixed set of parameters, the median rescue time is always shorter in the clique than in the star.

Despite these trends in the median times, the most remarkable feature of rescue time distributions is their high dispersion. As a result, the timing of tumor rescue is subject to a high degree of uncertainty, especially in star-like epistasis networks, what makes it essentially unpredictable.

### Epistasis-driven mutational profile and predictability of tumor rescue

A subtle yet relevant implication of the mutational tug-of-war model of cancer evolution is that, in the absence of epistasis, the fate of a precancerous lesion can be predicted with little uncertainty given the ratio of drivers to passengers. Indeed, it is possible to derive a critical ratio *R*_*c*_ such that cell populations will almost always progress to cancer if the driver-to-passenger ratio is greater than *R*_*c*_, and will remain stable or shrink otherwise (section 1.3 in S1 Text). Notably, although the number of drivers and passengers can change over time, their ratio in the population consistently remains below or above *R*_*c*_. That makes it feasible to predict the long-term behavior of a population based on its current mutational profile.

**Fig 6:**
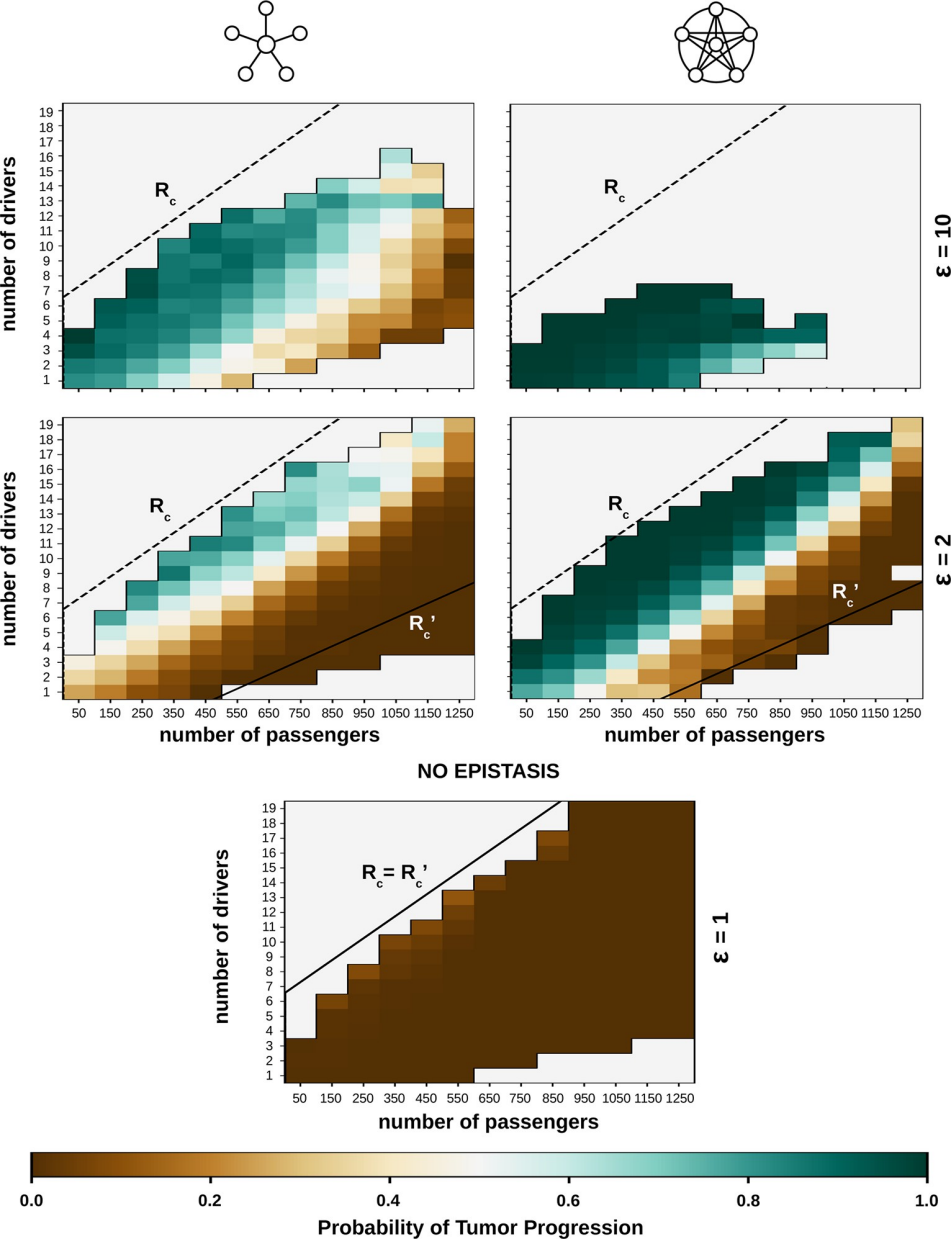
Predictability of tumor progression given the mutational profile. The number of drivers and passengers was collected at every time step for 1000 independent trajectories. For each combination with >10 observations, the figure indicates the fraction of trajectories that ended up doubling the initial size after 30,000 cell divisions. The dashed and solid lines correspond to the critical driver-to-passenger ratios (*R*_*c*_ and $R_{c}^{\prime }$) that determine the fate of the cell population before and after the trigger event. In the absence of epistasis $R_{c}=R_{c}^{\prime }$ (bottom plot). Parameter values: *μ* = 10^−8^, *s*_*d*_ = 0.07, *f*_*e*_ = 0.5, G = 70, rest of parameters as in Fig 2B.

The situation becomes more complicated if there is epistasis. In such cases, the strength and spread of epistasis determine a second critical ratio ($R_{c}^{\prime }$), such that $R_{c}^{\prime }<R_{c}$. Populations with driver-to-passenger ratios greater than *R*_*c*_ will still progress to cancer with high probability and those with ratios smaller than $R_{c}^{\prime }$ will not. However, the fate of populations with driver-to-passenger ratios between *R*_*c*_ and $R_{c}^{\prime }$ is less predictable. Simulations show that uncertainty increases with the strength of epistasis and depends on the structure of the epistasis network, with star-like networks being less predictable than clique-like ones (Fig 6).

**Fig 7:**
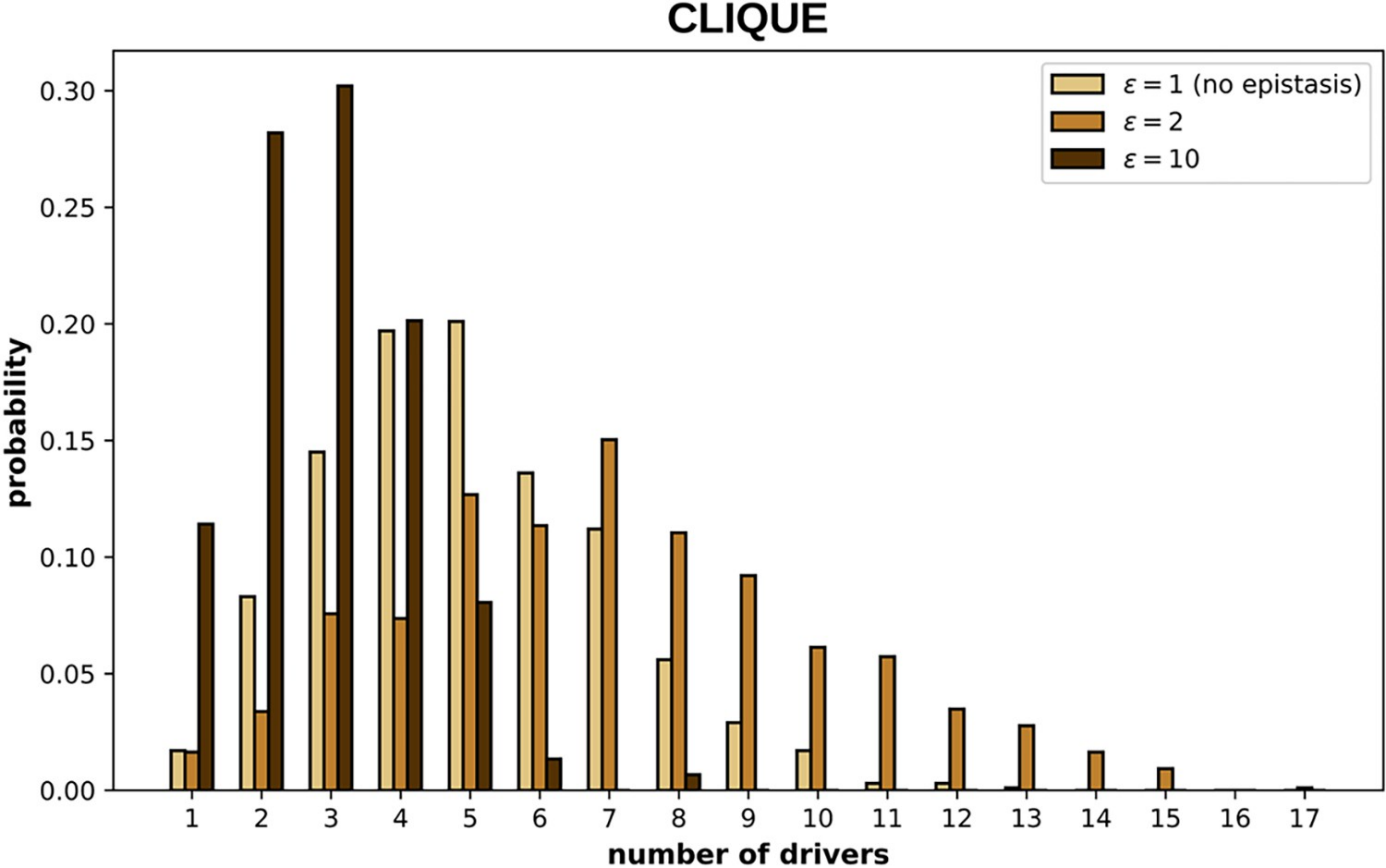
Number of drivers acquired by clones that do not progress to cancer. The distributions correspond to the maximum number of drivers observed along 1000 trajectories that did not double their initial size in 30,000 cell divisions, considering a clique-like epistasis network (see S2 Fig for a star-like network). Parameter values: *μ* = 5×10^−9^, *s*_*d*_ = 0.05, *f*_*e*_ = 0.5, G = 70, *ϵ* = 2, rest of parameters as in Fig 2B.

The existence of critical driver-to-passenger ratios for cancer progression implies that some cell populations can accumulate drivers during a lifetime without becoming aggressive tumors. This phenomenon is affected by epistasis in a complex way (Figs 7 and S3). More precisely, weak (but non-negligible) positive epistasis tends to increase the incidence of drivers in non-cancer clones, whereas strong positive epistasis has the opposite effect, both in clique- and star-like epistasis networks.

## Discussion

Positive epistasis among cancer drivers has been extensively described based on large-scale genomic studies and experimental evidence [26,28,31,32]. By including synergistic interactions in a mutational tug-of-war model of cancer evolution, we conducted an *in silico* analysis of the impact of epistasis on tumor progression. Computer simulations and analytical results suggest a crucial role of epistasis on the onset of cancer. More precisely, initially receding precancerous lesions can turn into rapidly growing tumors if trigger mutations appear in key genes of the epistasis network. Such evolutionary rescue of precancerous clones is only possible in a time window that depends on the mutation rate, the structure of the epistasis network, the strength of epistasis, and the properties of the local microenvironment that determine its carrying capacity. The existence of a limited time window for tumor rescue can contribute to explaining why potential drivers of cancer are often found in the genomes of non-neoplastic cells [14,15,17–23].

Our network-based framework for modeling epistasis is motivated by empirical data from cancer genomics and departs from classical statistical approaches, such as the rough Mt Fuji [51,52] and NK models [53]. Formally, the key to tumor rescue is the existence of one or a few key loci that, if mutated, substantially increase the fitness effect of mutations in a sufficiently large number of other loci. Most implementations of NK and rough Mt Fuji landscapes do not show such “one-to-many" asymmetry in the ratio of modifier to modified loci and, most importantly, use uncorrelated random variables to model the influence of a single locus on multiple other loci. Because of that, epistasis-driven rescue is highly unlikely in those models. On the other hand, the formal requisite for rescue is fulfilled by synergistic epistasis networks that contain one or several highly-connected genes, such as stars and cliques. In the case of star-like epistasis networks, mutations in a central cancer gene act as master switches that enable the tumorigenic effect of mutations in peripheral genes. Assuming that the order of fixation of driver events is strongly associated with their fitness effects [54,55], the tumor rescue model predicts a connection between a gene’s centrality in the epistasis network and the sequence of driver events. Thus, drivers in central genes are expected to reach fixation before drivers in peripheral genes. This trend has been empirically observed, for example, in endometrial cancer and glioblastoma, in which inactivation of *PTEN* and *TP53*, respectively, often precede driver mutations in secondary cancer genes [32,56,57]. Besides affecting the relative timing of driver events, synergistic interactions among drivers can modify the ratio of drivers to passengers and the distribution of driver mutations in clones that do not progress to cancer. In consequence, epistasis may constitute a substantial confounding factor when trying to infer tumor properties from mutational profiles.

The structure of epistasis networks has a major role in determining the probability and the timing of tumor rescue. Clique-like networks are more likely to trigger rescue because they contain more drivers that can act as potential triggers. In contrast, the rescue probability associated with star-like networks is always lower and completely dependent on the central gene. Our results suggest that the rescue of tumors with star-like epistasis networks is often restricted to populations with a mutator phenotype, which is consistent with the high mutation loads observed in many tumors [48]. Precancerous lesions with star-like epistasis networks tend to be less predictable than those with densely connected, clique-like epistasis networks, both in terms of the final outcome and the timing of rescue. Despite their simplicity, cliques and stars are relevant to the study of epistasis for two reasons: First, many real-world epistasis networks resemble clique- or star-like structures to some extent, depending on the asymmetry of their degree distributions. Second, even in cases in which the structural similarity between real-world networks and cliques or stars is not evident, the latter provide upper and lower bounds to the rescue probabilities, triggering times, and predictability.

Both positive and negative epistasis are pervasive in cancer. However, the structure of positive and negative epistasis networks is markedly distinct [32]. In many cancer types, groups of cancer genes involved in synergistic epistasis form separate communities of up to a dozen positively interacting genes, such as those represented in the left column of Fig 3A. In contrast, negative epistasis generally involves genes from different communities, leading to community-wise mutual exclusivity. As a result of this organization, negative epistasis can constrain the mutational trajectories of tumors by favoring a single community of cancer genes over the rest [32]. Thus, from the perspective of tumor rescue, the main effect of negative epistasis is to restrict the effective size of the epistasis network to a subset of cancer genes from the same community, reducing the rescue probability with respect to an alternative scenario in which cancer genes from different communities could simultaneously contribute to cancer progression.

In addition to driver mutations in cancer genes, other possible triggers of cancer include chromosomal rearrangements, mutations in regulatory regions, and changes in methylation [58–60]. These genetic and epigenetic alterations can be readily incorporated into the epistasis network as additional nodes with their own occurrence rates, fitness effects, and interaction profiles. As with regular drivers, their potential as triggers would be primarily determined by their centrality. In contrast, metabolic, inflammatory, hormonal, and other external changes that promote tumor growth [61–64] require a more careful consideration. First, some of those alterations can lead to direct (epistasis-independent) tumor rescue by increasing the carrying capacity of the tumor microenvironment above the critical threshold. Second, because synergistic gene-environment interactions can simultaneously operate on every mutated subclone, the probability of rescue associated with environmental factors may be greater than the epistasis-driven rescue probability associated with genetic or epigenetic alterations (for the same occurrence rate and interaction strength), especially in polyclonal cell populations.

The most relevant clinical implication of the mutational tug-of-war hypothesis for cancer evolution was that tumor regression can be induced by clinical interventions that reduce the carrying capacity of the tumor microenvironment below a critical size [43]. Our study generalizes that conclusion to scenarios with positive epistasis, although the critical carrying capacity in those cases may become much smaller. Moreover, interventions that initially succeed in reducing tumor size by increasing the deleterious effect of passengers may eventually fail due to epistasis-driven evolutionary rescue. In those cases, the identification of central genes in cancer epistasis networks can provide actionable targets for effective anticancer therapies.

## Methods

### Simulation of cancer evolution with positive epistasis

We conducted simulations to study the fate of a clonal precancerous lesion composed of *N*_0_ cells that initially harbor a single driver alteration. Although the model is formally described in the Results section, here we provide additional details about its computational implementation. The code for the simulation is provided as a supplementary file (S1 File). In our simulations, cells are grouped based on mutational profiles. Each mutational profile is characterized by the list of cancer genes that harbor driver mutations and the number of passenger alterations. (Recording the identity (not just the number) of cancer genes affected by driver mutations is necessary to calculate fitness in the presence of epistasis.) Mutational profiles are stored as a Python dictionary to facilitate information retrieval and incorporation of new genotypes throughout the simulation.

Following previous works [31,32,42,43], we assumed that the mutational target size for slightly deleterious passengers comprises *T*_*p*_ = 5×10^6^ sites and the mutational target size for drivers comprises *T*_*d*_ = 700 sites (70 cancer genes by 10 driver sites per cancer gene). The epistasis network is externally provided at the beginning of the simulation and it consists of a connected component with 70×*f*_*e*_ cancer genes and a set of 70×(1−*f*_*e*_) isolated cancer genes. Drivers in genes from the connected component have a fitness contribution *ϵs*_*d*_ if any of the neighbor genes in the network harbors a driver and *s*_*d*_ otherwise. Drivers in isolated cancer genes have always a fitness contribution *s*_*d*_. We assumed that once a driver mutation hits a cancer gene, additional drivers in the same gene will not contribute to fitness. We implemented that by reducing *T*_*d*_ in 10 sites for each new driver mutation and removing the genes that already harbor drivers from the list of “free” cancer genes. Each time that a new mutational profile appears in the population, its fitness value and the number of available driver sites is stored in the dictionary of mutational profiles.

Simulations start with a single mutational profile that contains *N*_0_ = 1000 cells with zero passengers and a single driver in one of the isolated cancer genes. At each generation, the offspring of each mutational profile is evaluated by sampling from a multinomial distribution with parameter *n* equal to the number of cells with that profile and probabilities $p=\left(\frac{D}{B+D},\frac{B}{B+D}\cdot {\left\{p_{k}\right\}}_{k=0}^{k_{max}}\right)$, where *p*_*k*_ is the probability of obtaining *k* mutations per division event, that follows a Poisson distribution with mean equal to *μ*(*T*_*d*_+*T*_*p*_) truncated at *k*_*max*_. We set a limit of *k*_*max*_ = 10 mutations per division per cell because the probability of obtaining larger values is extremely rare for the values of the parameters used in this study. If mutations occur, they are individually catalogued as passengers with probability $\frac{T_{p}}{T_{p}+T_{d}}$ or as drivers with probability $\frac{T_{d}}{T_{p}+T_{d}}$. In the case of drivers, they are randomly assigned to one of the available cancer genes. This procedure is iterated for all mutational profiles, subtracting the number of dead cells, adding the surviving daughter cells, and creating additional entries in a temporary dictionary if novel mutational profiles arise. Once all the profiles have been evaluated, the population is finally updated and a new generation can proceed.

Simulations run until one of the following conditions is reached: (i) the whole population becomes extinct (*N* = 0); (ii) the population doubles its initial size (*N* = 2*N*_0_) [42]; or (iii) the simulation reaches 30,000 generations.

### Positive epistasis networks

We simulated two extreme network topologies: a clique, in which all genes are connected with each other, and a star, in which a central cancer gene (the hub) is connected to a number of secondary (peripheral) cancer genes (Fig 3A). Because of their simplicity, the structure of cliques and stars is fully determined by their size. In this case, the size of the network is given by the product *C* = *Gf*_*e*_ (the total number of cancer genes multiplied by the fraction of genes that belong to the epistasis network) rounded to the closest integer. Cliques are built by taking *C* nodes and connecting all nodes with each other. Stars are built by taking a single node (the hub) and connecting it to the remaining *C*−1 nodes (the peripheral nodes).

Real-world epistasis networks typically display a modular structure with positive epistasis among the genes of the same module and negative epistasis among genes from different modules [32]. Due to such modular structure, tumor evolution leads to well-differentiated cancer subtypes, with each subtype characterized by mutations in one of the modules of the epistasis network. Therefore, to study the effect of positive epistasis on real-world networks, we started from a previously published collection of cancer epistasis networks [32], run the signed community detection tool SiMap [65] to identify the subsets of cancer genes associated with different cancer subtypes, and kept those modules that contained 5 or more cancer genes (Fig 3B left). Simulations were separately conducted for each cancer subtype. To that end, all cancer genes associated with the same cancer type but that do not belong to the subtype-specific epistasis network were considered as disconnected nodes.

To compare the rescue probability and trigger times of empirical epistasis networks and simple model networks (Fig 3B) we proceeded as follows. For each cancer subtype network, we collected the total number of cancer genes identified in that cancer subtype (*G*_*i*_), the number of cancer genes that belong to the connected component of the network (*C*_*i*_), and the connectivity of the hub (*K*_*i*_). The corresponding star network was built by connecting a single node (the hub) to *K*_*i*_ peripheral nodes. The regular network was built as a regular ring with size *C*_*i*_ and connectivity *K*_*i*_. Because regular rings can only have an even connectivity, in those cases in which the empirical *K*_*i*_ was an odd number we built a regular ring of connectivity *K*_*i*_−1 and then sequentially assigned the remaining edges from randomly selected nodes to their [(*K*_*i*_+1)/2]-th nearest neighbors while ensuring that no node had connectivity greater than *K*_*i*_.

### Quantification of tumor rescue through simulations

To assess the probability of evolutionary rescue mediated by epistasis, we set parameter values within realistic biological ranges (Table 1), so that the initial cell population tends to recede in the absence of epistasis. We then explored the effect of varying the mutation rate (*μ*), driver fitness effect (*s*_*d*_), epistasis strength (*ϵ*), fraction of genes subject to epistasis (*f*_*e*_), and network topology. For each parameter combination, we run 1000 simulations and classified the outcome of each simulation as tumor rescue if the size of the cell population had doubled after 30,000 generations. The cancer probability was calculated as the fraction of simulations in which rescue took place.

### Approximate analytical study of tumor rescue

Here we summarize the main expressions obtained from an approximate analytical study of the model. To obtain these expressions, we assumed that mutation accumulation is a deterministic process (except for the occurrence and fixation of the trigger driver) and that the population size is equal to the effective carrying capacity of the system (given by the product *BN*_0_) at any time. The detailed derivation of these expressions can be found in the S1 Text.

Approximate expressions for the tumor rescue probability can be derived for star- and clique-like epistasis networks by taking advantage of their particularly simple structures. In the star, the first driver mutation in the hub modifies the fitness effect of drivers in any other gene of the network. In the clique, the first driver mutation in any gene of the network modifies the fitness effect of drivers in all other genes. We call these first mutations *trigger drivers* because they amplify the effect of other drivers in the cancer gene network. An important property of star- and clique-like epistasis networks is that the critical population size that determines the fate of the cell population can take two values, *N*_*c*_ and $N_{c}^{\prime }$, depending on the mutation landscape. The pre-trigger critical size *N*_*c*_ applies to populations that lack fixed driver mutations in the hub (for star-like epistasis networks) or in any gene of the network (for clique-like epistasis networks). In those circumstances, all drivers have the same fitness effect *s*_*d*_ and the critical population size is given by $N_{c}=\frac{T_{p}s_{p}(1+s_{d})}{T_{d}{s_{d}}^{2}}$. The post-trigger critical size $N_{c}^{\prime }$ applies to populations that harbor driver mutations in the hub (for star-like epistasis networks) or in any gene of the network (for clique-like epistasis networks). After the trigger driver reaches fixation, the critical population size becomes $N_{c}^{\prime }=N_{c}{\left[1+f_{e}(\epsilon^{2}\frac{1+s_{d}}{1+\epsilon s_{d}}-1)\right]}^{-1}$ (section 1.2 in S1 Text). In a scenario of population decay, tumor rescue occurs if the trigger driver appears before the population size falls below $N_{c}^{\prime }$. Accordingly, if we define *t*_*c*_ as the critical time at which a decaying population reaches the size $N_{c}^{\prime }$, the rescue probability becomes $P=1-e^{-D(t_{c})}$, where $D(t_{c})=\mu T_{D}\sum_{t=1}^{t_{c}}N(t)q_{D}(t)$ is the expected number of trigger drivers that get fixed in the population before the critical rescue time, *T*_*D*_ is the mutational target size associated with trigger drivers (the hub in the star and any gene of the network the clique), and *q*_*D*_(*t*) is the fixation probability of a trigger driver that appeared at time *t*. In the clique, *q*_*D*_(*t*) can be reasonably approximated as the fixation probability of a slightly beneficial mutation in the limit of large population size, that is *q*_*D*_(*t*)≈*s*_*d*_/(1+*s*_*d*_). In the star, the fixation probability of the trigger driver depends on the pre-existence of driver mutations in peripheral cancer genes, which results in a more convoluted expression, $q_{D}(t)\approx 1-e^{-\xi (t)}\left[\frac{1}{1+s_{d}}-\frac{1}{1+\epsilon s_{d}}(1-e^{\frac{\xi (1+s_{d})}{1+\epsilon s_{d}}})\right]$ (section 1.4 in S1 Text). In that expression, the factor *ξ*(*t*) represents the expected number of drivers in peripheral genes and is given by $\xi (t)=\frac{\mu T_{d}f_{e}s_{d}}{1+s_{d}}\sum_{k=0}^{t-1}N(k)$. As expected, in the absence of drivers in peripheral genes, the fixation probabilities of trigger drivers in the star and the clique are the same.

## Supporting information

S1 Text Supplementary notes and mathematical derivations regarding the approximate analytical study of the model. (PDF)

S1 FigComparison of the analytical probabilities of tumor progression and the probabilities obtained in simulations.The plot compares the rescue probabilities obtained for all parameter values explored in Figs 4 and S2. The overall coefficient of determination, calculated by taking the values from simulations as the “true” ones, is *R*^2^ = 0.987 (*n* = 1056). The coefficients of determination for each dataset are: clique with *ϵ* = 2, *R*^2^ = 0.962; clique with *ϵ* = 10, *R*^2^ = 0.994; star with *ϵ* = 2, *R*^2^ = 0.978; star with *ϵ* = 10, *R*^2^ = 0.984. The lower accuracy of the analytical approximation in cliques with *ϵ* = 2 results from the occasional rescue of tumors after the critical time (see section 1.4.3 in S1 Text). Such “late” rescue can occur if stochastic fluctuations in the rate at which drivers accumulate lead to fixation of an enhanced driver shortly after the trigger. These stochastic effects are more relevant in conditions in which the trigger driver only produces a modest increase in the population size (Δ*N*_*D*_, see section 1.4.2 in S1 Text) and the critical population size after rescue, $N_{c}^{\prime }$, is relatively close to *N*_*c*_. Both conditions are especially met by cliques (due to the absence of preexistent drivers in the epistasis network, that leads to relatively small Δ*N*_*D*_) with low epistasis factors (that lead to modest decreases in $N_{c}^{\prime }$). Even in those cases, despite the systematic bias in analytical approximations, the analytical expression can distinguish between scenarios of high and low rescue probabilities (only 1.3% of the data points have rescue probability >0.5 in the simulations and <0.5 in the analytical expression or vice versa).(TIF)

S2 Fig Tumor rescue is facilitated by high mutation rates and strong epistasis. (A) Analytical probability of tumor progression as a function of network structure, mutation rate (*μ*), driver fitness effect (*s*_*d*_), fraction of genes subject to epistasis (*f*_*e*_), and strength of epistasis (*ϵ*).
(TIF)

S3 Fig Number of drivers acquired by clones that do not progress to cancer. The distributions correspond to the maximum number of drivers observed along 1000 trajectories that did not double their initial size in 30000 cell divisions, considering a star-like epistasis network (see Fig 7 for a clique-like network). Parameter values: *μ* = 5×10^−9^, *s*_*d*_ = 0.05, *f*_*e*_ = 0.5, *ϵ* = 2, rest of parameters as in Fig 3.
(TIF)

S1 File Code for simulations. Python code used to perform the stochastic simulations of the model.
(PY)

## References

[1] Hanahan D, Weinberg RA. The hallmarks of cancer. Cell. 2000;100(1):57–70. doi: 10.1016/s0092-8674(00)81683-9 . 10647931

[2] Stratton MR, Campbell PJ, Futreal PA. The cancer genome. Nature. 2009;458(7239):719–24. doi: 10.1038/nature07943 ; PubMed Central PMCID: PMC2821689. 19360079 PMC2821689

[3] Yates LR, Campbell PJ. Evolution of the cancer genome. Nature reviews Genetics. 2012;13(11):795–806. doi: 10.1038/nrg3317 ; PubMed Central PMCID: PMC3666082. 23044827 PMC3666082

[4] Merlo LM, Pepper JW, Reid BJ, Maley CC. Cancer as an evolutionary and ecological process. Nature reviews Cancer. 2006;6(12):924–35. doi: 10.1038/nrc2013 . 17109012

[5] Weghorn D, Sunyaev S. Bayesian inference of negative and positive selection in human cancers. Nat Genet. 2017;49(12):1785–8. doi: 10.1038/ng.3987 . 29106416

[6] Martincorena I, Raine KM, Gerstung M, Dawson KJ, Haase K, Van Loo P, et al. Universal Patterns of Selection in Cancer and Somatic Tissues. Cell. 2017. doi: 10.1016/j.cell.2017.09.042 . 29056346 PMC5720395

[7] Banyai L, Trexler M, Kerekes K, Csuka O, Patthy L. Use of signals of positive and negative selection to distinguish cancer genes and passenger genes. Elife. 2021;10. doi: 10.7554/eLife.59629 ; PubMed Central PMCID: PMC7877913. 33427197 PMC7877913

[8] Good BH, Desai MM. Deleterious passengers in adapting populations. Genetics. 2014;198(3):1183–208. doi: 10.1534/genetics.114.170233 ; PubMed Central PMCID: PMC4224160. 25194161 PMC4224160

[9] Pybus OG, Rambaut A, Belshaw R, Freckleton RP, Drummond AJ, Holmes EC. Phylogenetic evidence for deleterious mutation load in RNA viruses and its contribution to viral evolution. Molecular biology and evolution. 2007;24(3):845–52. doi: 10.1093/molbev/msm001 . 17218639

[10] Covert AW, 3rd, Lenski RE, Wilke CO, Ofria C. Experiments on the role of deleterious mutations as stepping stones in adaptive evolution. Proceedings of the National Academy of Sciences of the United States of America. 2013;110(34):E3171–8. doi: 10.1073/pnas.1313424110 ; PubMed Central PMCID: PMC3752215. 23918358 PMC3752215

[11] McFarland CD, Yaglom JA, Wojtkowiak JW, Scott JG, Morse DL, Sherman MY, et al. The Damaging Effect of Passenger Mutations on Cancer Progression. Cancer research. 2017;77(18):4763–72. doi: 10.1158/0008-5472.CAN-15-3283-T ; PubMed Central PMCID: PMC5639691. 28536279 PMC5639691

[12] Budzinska MA, Tu T, d’Avigdor WM, McCaughan GW, Luciani F, Shackel NA. Accumulation of Deleterious Passenger Mutations Is Associated with the Progression of Hepatocellular Carcinoma. PLoS One. 2016;11(9):e0162586. doi: 10.1371/journal.pone.0162586 ; PubMed Central PMCID: PMC5025244. 27631787 PMC5025244

[13] Tilk S, Tkachenko S, Curtis C, Petrov DA, McFarland CD. Most cancers carry a substantial deleterious load due to Hill-Robertson interference. Elife. 2022;11. doi: 10.7554/eLife.67790 ; PubMed Central PMCID: PMC9499534. 36047771 PMC9499534

[14] Mutter GL, Monte NM, Neuberg D, Ferenczy A, Eng C. Emergence, involution, and progression to carcinoma of mutant clones in normal endometrial tissues. Cancer research. 2014;74(10):2796–802. doi: 10.1158/0008-5472.CAN-14-0108 ; PubMed Central PMCID: PMC4058864. 24662919 PMC4058864

[15] Xie M, Lu C, Wang J, McLellan MD, Johnson KJ, Wendl MC, et al. Age-related mutations associated with clonal hematopoietic expansion and malignancies. Nature medicine. 2014;20(12):1472–8. doi: 10.1038/nm.3733 ; PubMed Central PMCID: PMC4313872. 25326804 PMC4313872

[16] Martincorena I, Campbell PJ. Somatic mutation in cancer and normal cells. Science. 2015;349(6255):1483–9. doi: 10.1126/science.aab4082 . 26404825

[17] Martincorena I, Roshan A, Gerstung M, Ellis P, Van Loo P, McLaren S, et al. Tumor evolution. High burden and pervasive positive selection of somatic mutations in normal human skin. Science. 2015;348(6237):880–6. doi: 10.1126/science.aaa6806 ; PubMed Central PMCID: PMC4471149. 25999502 PMC4471149

[18] Anglesio MS, Papadopoulos N, Ayhan A, Nazeran TM, Noe M, Horlings HM, et al. Cancer-Associated Mutations in Endometriosis without Cancer. The New England journal of medicine. 2017;376(19):1835–48. doi: 10.1056/NEJMoa1614814 ; PubMed Central PMCID: PMC5555376. 28489996 PMC5555376

[19] Martincorena I, Fowler JC, Wabik A, Lawson ARJ, Abascal F, Hall MWJ, et al. Somatic mutant clones colonize the human esophagus with age. Science. 2018;362(6417):911–7. doi: 10.1126/science.aau3879 ; PubMed Central PMCID: PMC6298579. 30337457 PMC6298579

[20] Risques RA, Kennedy SR. Aging and the rise of somatic cancer-associated mutations in normal tissues. PLoS genetics. 2018;14(1):e1007108. doi: 10.1371/journal.pgen.1007108 ; PubMed Central PMCID: PMC5754046 report: 1) He is a paid consultant and equity holder of TwinStrand BioSciences Inc; 2) he has a submitted two patent application concerning methods to detect low frequency mutations with a priority date of 3/23/17; 3) he is the recipient of patent royalty payments concerning Duplex Sequencing; and 4) he has a research program funded by three grants from the Department of Defense and one from the National Institute of Justice. Rosa Ana Risques has read the journal’s policy and is declaring that her research program is funded by two grants from the National Institutes of Health, one of which is a SBIR grant with TwinStrand BioSciences, Inc., a grant from the Rivkin Center for Ovarian Cancer, and a grant from the Mary Kay Foundation. 29300727 PMC5754046

[21] Moore L, Leongamornlert D, Coorens THH, Sanders MA, Ellis P, Dentro SC, et al. The mutational landscape of normal human endometrial epithelium. Nature. 2020;580(7805):640–6. doi: 10.1038/s41586-020-2214-z . 32350471

[22] Piedrafita G, Fernández LC, Real FX. Mutations in Non-Tumoral Human Urothelium: Disease Prelude or Epilogue? Bladder Cancer. 2020;6(3):249–52. doi: 10.3233/blc-200363

[23] Colom B, Herms A, Hall MWJ, Dentro SC, King C, Sood RK, et al. Mutant clones in normal epithelium outcompete and eliminate emerging tumours. Nature. 2021;598(7881):510–4. doi: 10.1038/s41586-021-03965-7 ; PubMed Central PMCID: PMC7612642. 34646013 PMC7612642

[24] Persi E, Wolf YI, Horn D, Ruppin E, Demichelis F, Gatenby RA, et al. Mutation-selection balance and compensatory mechanisms in tumour evolution. Nature reviews Genetics. 2021;22(4):251–62. doi: 10.1038/s41576-020-00299-4 . 33257848

[25] Park S, Lehner B. Cancer type-dependent genetic interactions between cancer driver alterations indicate plasticity of epistasis across cell types. Mol Syst Biol. 2015;11(7):824. doi: 10.15252/msb.20156102 ; PubMed Central PMCID: PMC4547852. 26227665 PMC4547852

[26] Zhang H, Deng Y, Zhang Y, Ping Y, Zhao H, Pang L, et al. Cooperative genomic alteration network reveals molecular classification across 12 major cancer types. Nucleic acids research. 2017;45(2):567–82. doi: 10.1093/nar/gkw1087 ; PubMed Central PMCID: PMC5314758. 27899621 PMC5314758

[27] Park S, Supek F, Lehner B. Higher order genetic interactions switch cancer genes from two-hit to one-hit drivers. Nature communications. 2021;12(1):7051. doi: 10.1038/s41467-021-27242-3 ; PubMed Central PMCID: PMC8642467. 34862370 PMC8642467

[28] Wang X, Fu AQ, McNerney ME, White KP. Widespread genetic epistasis among cancer genes. Nature communications. 2014;5:4828. doi: 10.1038/ncomms5828 . 25407795

[29] Land H, Parada LF, Weinberg RA. Tumorigenic conversion of primary embryo fibroblasts requires at least two cooperating oncogenes. Nature. 1983;304(5927):596–602. doi: 10.1038/304596a0 . 6308472

[30] Rajagopalan H, Bardelli A, Lengauer C, Kinzler KW, Vogelstein B, Velculescu VE. Tumorigenesis: RAF/RAS oncogenes and mismatch-repair status. Nature. 2002;418(6901):934. doi: 10.1038/418934a . 12198537

[31] Mina M, Raynaud F, Tavernari D, Battistello E, Sungalee S, Saghafinia S, et al. Conditional Selection of Genomic Alterations Dictates Cancer Evolution and Oncogenic Dependencies. Cancer cell. 2017;32(2):155–68 e6. doi: 10.1016/j.ccell.2017.06.010 . 28756993

[32] Iranzo J, Gruenhagen G, Calle-Espinosa J, Koonin EV. Pervasive conditional selection of driver mutations and modular epistasis networks in cancer. Cell Rep. 2022;40(8):111272. doi: 10.1016/j.celrep.2022.111272 . 36001960

[33] Iranzo J, Martincorena I, Koonin EV. Cancer-mutation network and the number and specificity of driver mutations. Proceedings of the National Academy of Sciences of the United States of America. 2018;115(26):E6010–E9. doi: 10.1073/pnas.1803155115 ; PubMed Central PMCID: PMC6042135. 29895694 PMC6042135

[34] Zhao Y, Murciano-Goroff YR, Xue JY, Ang A, Lucas J, Mai TT, et al. Diverse alterations associated with resistance to KRAS(G12C) inhibition. Nature. 2021;599(7886):679–83. doi: 10.1038/s41586-021-04065-2 ; PubMed Central PMCID: PMC8887821. 34759319 PMC8887821

[35] Yeang CH, McCormick F, Levine A. Combinatorial patterns of somatic gene mutations in cancer. FASEB J. 2008;22(8):2605–22. doi: 10.1096/fj.08-108985 . 18434431

[36] Jerby-Arnon L, Pfetzer N, Waldman YY, McGarry L, James D, Shanks E, et al. Predicting cancer-specific vulnerability via data-driven detection of synthetic lethality. Cell. 2014;158(5):1199–209. doi: 10.1016/j.cell.2014.07.027 . 25171417

[37] Kim YA, Madan S, Przytycka TM. WeSME: uncovering mutual exclusivity of cancer drivers and beyond. Bioinformatics. 2017;33(6):814–21. doi: 10.1093/bioinformatics/btw242 ; PubMed Central PMCID: PMC5888950. 27153670 PMC5888950

[38] Matlak D, Szczurek E. Epistasis in genomic and survival data of cancer patients. PLoS computational biology. 2017;13(7):e1005626. doi: 10.1371/journal.pcbi.1005626 ; PubMed Central PMCID: PMC5517071. 28678836 PMC5517071

[39] Srihari S, Singla J, Wong L, Ragan MA. Inferring synthetic lethal interactions from mutual exclusivity of genetic events in cancer. Biol Direct. 2015;10:57. doi: 10.1186/s13062-015-0086-1 ; PubMed Central PMCID: PMC4590705. 26427375 PMC4590705

[40] Wappett M, Dulak A, Yang ZR, Al-Watban A, Bradford JR, Dry JR. Multi-omic measurement of mutually exclusive loss-of-function enriches for candidate synthetic lethal gene pairs. BMC genomics. 2016;17:65. doi: 10.1186/s12864-016-2375-1 ; PubMed Central PMCID: PMC4717622. 26781748 PMC4717622

[41] Wilkins JF, Cannataro VL, Shuch B, Townsend JP. Analysis of mutation, selection, and epistasis: an informed approach to cancer clinical trials. Oncotarget. 2018;9(32):22243–53. doi: 10.18632/oncotarget.25155 ; PubMed Central PMCID: PMC5976461. 29854275 PMC5976461

[42] McFarland CD, Korolev KS, Kryukov GV, Sunyaev SR, Mirny LA. Impact of deleterious passenger mutations on cancer progression. Proceedings of the National Academy of Sciences of the United States of America. 2013;110(8):2910–5. doi: 10.1073/pnas.1213968110 ; PubMed Central PMCID: PMC3581883. 23388632 PMC3581883

[43] McFarland CD, Mirny LA, Korolev KS. Tug-of-war between driver and passenger mutations in cancer and other adaptive processes. Proceedings of the National Academy of Sciences of the United States of America. 2014;111(42):15138–43. doi: 10.1073/pnas.1404341111 ; PubMed Central PMCID: PMC4210325. 25277973 PMC4210325

[44] Tomasetti C, Vogelstein B. Cancer etiology. Variation in cancer risk among tissues can be explained by the number of stem cell divisions. Science. 2015;347(6217):78–81. doi: 10.1126/science.1260825 ; PubMed Central PMCID: PMC4446723. 25554788 PMC4446723

[45] Gao Z, Wyman MJ, Sella G, Przeworski M. Interpreting the Dependence of Mutation Rates on Age and Time. PLoS Biol. 2016;14(1):e1002355. doi: 10.1371/journal.pbio.1002355 ; PubMed Central PMCID: PMC4711947. 26761240 PMC4711947

[46] Knudson AG. Two genetic hits (more or less) to cancer. Nature reviews Cancer. 2001;1(2):157–62. doi: 10.1038/35101031 . 11905807

[47] Potten CS, Kellett M, Rew DA, Roberts SA. Proliferation in human gastrointestinal epithelium using bromodeoxyuridine in vivo: data for different sites, proximity to a tumour, and polyposis coli. Gut. 1992;33(4):524–9. doi: 10.1136/gut.33.4.524 ; PubMed Central PMCID: PMC1374071. 1316306 PMC1374071

[48] Loeb LA, Bielas JH, Beckman RA. Cancers exhibit a mutator phenotype: clinical implications. Cancer research. 2008;68(10):3551–7; discussion 7. doi: 10.1158/0008-5472.CAN-07-5835 . 18483233

[49] Bozic I, Antal T, Ohtsuki H, Carter H, Kim D, Chen S, et al. Accumulation of driver and passenger mutations during tumor progression. Proceedings of the National Academy of Sciences of the United States of America. 2010;107(43):18545–50. doi: 10.1073/pnas.1010978107 ; PubMed Central PMCID: PMC2972991. 20876136 PMC2972991

[50] Korolev KS, Xavier JB, Gore J. Turning ecology and evolution against cancer. Nature reviews Cancer. 2014;14(5):371–80. doi: 10.1038/nrc3712 . 24739582 PMC13213539

[51] Aita T, Uchiyama H, Inaoka T, Nakajima M, Kokubo T, Husimi Y. Analysis of a local fitness landscape with a model of the rough Mt. Fuji-type landscape: application to prolyl endopeptidase and thermolysin. Biopolymers. 2000;54(1):64–79. doi: 10.1002/(SICI)1097-0282(200007)54:1&lt;64::AID-BIP70&gt;3.0.CO;2-R . 10799982

[52] Neidhart J, Szendro IG, Krug J. Adaptation in tunably rugged fitness landscapes: the rough Mount Fuji model. Genetics. 2014;198(2):699–721. doi: 10.1534/genetics.114.167668 ; PubMed Central PMCID: PMC4196622. 25123507 PMC4196622

[53] Kauffman SA. The origins of order: self-organization and selection in evolution. New York: Oxford University Press; 1993. xviii, 709 p. p.

[54] Paterson C, Clevers H, Bozic I. Mathematical model of colorectal cancer initiation. Proceedings of the National Academy of Sciences of the United States of America. 2020;117(34):20681–8. doi: 10.1073/pnas.2003771117 ; PubMed Central PMCID: PMC7456111. 32788368 PMC7456111

[55] Gerstung M, Eriksson N, Lin J, Vogelstein B, Beerenwinkel N. The temporal order of genetic and pathway alterations in tumorigenesis. PLoS One. 2011;6(11):e27136. doi: 10.1371/journal.pone.0027136 ; PubMed Central PMCID: PMC3206070 Dr. Eriksson joined 23andMe after finishing the work on the present manuscript. It is unrelated to his current affiliation and no competing interest exists. 22069497 PMC3206070

[56] Levine RL, Cargile CB, Blazes MS, van Rees B, Kurman RJ, Ellenson LH. PTEN mutations and microsatellite instability in complex atypical hyperplasia, a precursor lesion to uterine endometrioid carcinoma. Cancer research. 1998;58(15):3254–8. . 9699651

[57] Zhang S, Yu D. PI(3)king apart PTEN’s role in cancer. Clin Cancer Res. 2010;16(17):4325–30. doi: 10.1158/1078-0432.CCR-09-2990 . 20622047

[58] Bhattacharya A, Bense RD, Urzua-Traslavina CG, de Vries EGE, van Vugt M, Fehrmann RSN. Transcriptional effects of copy number alterations in a large set of human cancers. Nature communications. 2020;11(1):715. doi: 10.1038/s41467-020-14605-5 ; PubMed Central PMCID: PMC7002723 from Sanofi, Daiichi, Sankyo, NSABP, Pfizer and Merck, and institutional financial support for clinical trials or contracted research from Amgen, Genentech, Roche, AstraZeneca, Synthon, Nordic Nanovector, G1 Therapeutics, Bayer, Chugai Pharma, CytomX Therapeutics and Radius Health, all unrelated to the submitted work. All other authors declare no competing interests. 32024838 PMC7002723

[59] Sharma S, Kelly TK, Jones PA. Epigenetics in cancer. Carcinogenesis. 2010;31(1):27–36. doi: 10.1093/carcin/bgp220 ; PubMed Central PMCID: PMC2802667. 19752007 PMC2802667

[60] Baylin SB, Jones PA. Epigenetic Determinants of Cancer. Cold Spring Harb Perspect Biol. 2016;8(9). doi: 10.1101/cshperspect.a019505 ; PubMed Central PMCID: PMC5008069. 27194046 PMC5008069

[61] Greten FR, Grivennikov SI. Inflammation and Cancer: Triggers, Mechanisms, and Consequences. Immunity. 2019;51(1):27–41. doi: 10.1016/j.immuni.2019.06.025 ; PubMed Central PMCID: PMC6831096. 31315034 PMC6831096

[62] Coller HA. Is cancer a metabolic disease? Am J Pathol. 2014;184(1):4–17. doi: 10.1016/j.ajpath.2013.07.035 ; PubMed Central PMCID: PMC3873478. 24139946 PMC3873478

[63] Dang CV. Links between metabolism and cancer. Genes Dev. 2012;26(9):877–90. doi: 10.1101/gad.189365.112 ; PubMed Central PMCID: PMC3347786. 22549953 PMC3347786

[64] Key TJ. Hormones and cancer in humans. Mutat Res. 1995;333(1–2):59–67. doi: 10.1016/0027-5107(95)00132-8 . 8538637

[65] Esmailian P, Jalili M. Community Detection in Signed Networks: the Role of Negative ties in Different Scales. Scientific reports. 2015;5:14339. doi: 10.1038/srep14339 ; PubMed Central PMCID: PMC4585820. 26395815 PMC4585820

