# Supplementary material for: Synergistic epistasis among cancer drivers can rescue early tumors from the accumulation of deleterious passengers
Source: PLoS Comput Biol. 2024 Apr 30;20(4):e1012081. doi: 10.1371/journal.pcbi.1012081 (PMC11087069; doi:10.1371/journal.pcbi.1012081)
Supplement: S1 Text — (PDF) [file pcbi.1012081.s001.pdf]

S1 Text: Supplementary Information

Synergistic epistasis among cancer drivers can rescue early tumors from the accumulation of deleterious passengers.

C. Alejandre, J. Calle-Espinosa, J. Iranzo

Contents

1 Approximate analytical study of the model **2**
1.1 General considerations . . . 2
1.2 Critical population size . . . 4
1.3 Critical driver-to-passenger ratio . . . 5
1.4 Tumor rescue . . . 6
1.4.1 Population size immediately before triggering . . . 7
1.4.2 Population size immediately after triggering . . . 7
1.4.3 Critical trigger time . . . 8
1.4.4 Rescue probability . . . 9
1.5 Critical rescue time for population doubling . . . 10

1 Approximate analytical study of the model

In this section we present an approximate analytical study of the model described in the main text, following the same general lines of reasoning as in the original works by McFarland et al. [1, 2]. The expressions derived in this section make heavy use of classical approximations from statistic physics (*e.g.*, timescale separation) and population genetics (*e.g.*, the fixation probability of beneficial and slightly deleterious mutations). The appropriateness and validity of these approximations is thoroughly discussed by McFarland et al. [1,2] and supported by simulations (S1 Fig). We have attempted to provide a sufficient level of detail to allow the reader to follow the main derivations. In any case, we refer the reader to the original articles for further explanations.

1.1 General considerations

To complement the simulations described in the main text, we developed a series of approximate analytical expressions that helped us gain an intuitive understanding of the factors that determine tumor rescue in the presence of positive epistasis. To obtain such expressions, we adopted a number of approximations that facilitate analytical calculations. Most of the approximations have been previously justified in the context of a simpler model without epistasis [1, 2]. However, since they concern general aspects of the modeling framework (for example, the existence of a critical size for tumor progression), they are equally applicable to a scenario with epistasis. Henceforth, we adopt the following assumptions and approximations:

- Despite the simulations operate in discrete generations and deal with discrete cell numbers, for our analytical calculations we assume that the dynamics of large populations can be approximated as a deterministic process with continuous time and population size. If the number of mutations remains stable, the population dynamics is described by the following differential equation

$$\dot{N} = N\left(B - D(N)\right) \tag{1}$$

  where N is the population size, B is the cell duplication rate, and $D(N)$ is the cell death rate.

- To maintain consistency with the simluations, we rescale time by the duplication rate B. This is equivalent to measuring time in units of cell generations. Because cell division rates vary across tissues and empirical mutation frequencies are better predicted by the number of divisions than by absolute time [3, 4], measuring time in terms of cell generations is also a more appropriate choice from a biological perspective. The change of time scale leads to the following equation for the population dynamics:

$$\dot{N} = N\left(1 - \frac{D(N)}{B}\right) \tag{2}$$

  Note that the dynamics only depend on the ratio between death and duplication rates. Therefore, both rates can be jointly scaled by any constant factor without changing the dynamics.

- As described in the main text, cells can harbor different numbers of passenger mutations (n_p), “regular” drivers (n_d) whose fitness effects are not affected by epistasis given the current mutational background, and “enhanced” drivers (n_ϵ) whose fitness effects are augmented as a result of synergistic interactions with other drivers. A driver mutation affecting a particular gene g is enhanced if there is at least another driver mutation affecting any of the genes connected to g in the epistasis network. We assume that mutations modify the fitness of the tumor by a multiplicative factor, such that

$$B = \frac{(1+s_d)^{n_d}(1+\epsilon s_d)^{n_\epsilon}}{(1+s_p)^{n_p}} \tag{3}$$

where s_d and s_p are the fitness effects of regular drivers and passengers, respectively, and ϵ is a multiplicative factor that accounts for the effect of synergistic epistasis on the fitness effect of drivers. Moreover, we assume a density-dependent death rate

$$D = \frac{N}{N_0} \tag{4}$$

where N_0 represents the carrying capacity in the absence of mutations. Combining eqns. 2 and 4, we obtain the following logistic equation for the population dynamics:

$$\dot{N} = N\left(1 - \frac{N}{BN_0}\right) \tag{5}$$

From eqns. 3 and 5 it becomes clear that mutations affect the system by modifying its effective carrying capacity.

- Based on eqn. 5, the equilibrium population size (N^*) with constant mutation load is equal to

  $$N^* = BN_0 \tag{6}$$

  Eqn. 5 also allows estimating the duration of the transient before that equilibrium size is reached. For example, starting from 1 cell, the population reaches 99.9% of the initial carrying capacity (set to $N_0 = 1000$) in just 14 generations. Considering the range of fitness effects that we explored in the simulations (Table 1 in the main text) and their contribution to B, when a new mutation reaches fixation in the population the new equilibrium size is typically reached in very few generations. As an upper bound, let us consider the extreme case with $s_d = 0.1$, $\epsilon = 10$, and suppose that the fixation of an enhanced driver in the whole population increases BN_0 from 1000 to 2000 (this is the largest possible change compatible with the range of parameters and stop criteria). According to eqn. 5, the new equilibrium (within a tolerance of 0.001%) will be reached in only 7 generations. In the case of passengers, the transient lasts < 1 generation. In contrast, the typical waiting time for the occurrence and fixation of passengers is approximately 20 generations (with $\mu = 10^{-8}$ and $T_p = 5 \times 10^6$) and around $10 - 100$ times higher for drivers (see below for details). Because of this difference in timescales, we assume that every time that a new mutation reaches fixation, the population size instantaneously adjusts to the new equilibium value given by eqn. 6. (Note that this approximation only concerns analytical calculations: the simulations explicitly consider each cell in the population and their stochastic dynamics.)

- We assume that driver and passenger mutations occur during cell division. This assumtion is justified by the empirical observation that cancer risk is proportional to the number of stem cell divisions in different tissues [3]. The number of drivers and passengers acquired by each daughter cell follows a Poisson distribution with mean μT_d for drivers and μT_p for passengers, where μ is the mutation rate per site per cell per generation, T_d is the number of possible driver sites per cell, and T_p is the number of possible passenger sites per cell. As indicated in Table 1 (main text), $T_p \gg T_d$.

- Driver mutations are randomly assigned to cancer genes. We assume that there are G cancer genes that equally contribute to the total number of driver sites. Only a fraction f_e of the cancer genes are connected to the epistasis network. In consequence, only a fraction f_e of all possible drivers can be enhanced. We study two classes of networks that contain genes that, if mutated, modify the fitness effect of drivers in any other gene of the network. Those are clique-like networks, in which every gene is connected to each other; and star-like networks, in which a single central gene (the hub) is connected to all the other (peripheral) genes. In the clique, any driver mutation will trigger the effects of epistasis, modifying the fitness effects of drivers in any other gene of the network. In

contrast, in the star, only mutations in the hub can act as triggers. The existence of a single trigger event implies that we can characterize the global state of clique- and star-like epistasis networks as "active" (if the trigger has occured) or "inactive" (before the trigger occurs). Note that this is not generally possible in more complex networks.

- Although the simulations consider the fully stochastic nature of the mutation process, for the analytical calculations we assume that accumulation of drivers and passengers can be approximated as deterministic processes, whose rates are given by the product of the population-level mutation rates and the fixation probabilities. This approximation is justified by numerical simulations, as shown by McFarland et al. [1, 2]. It follows from the previous point that the population-level mutation rates are $\mu(1-f_e)T_dN$ for regular drivers, $\mu f_e T_d N$ for enhanced drivers, and $\mu T_p N$ for passengers. To estimate the probability of fixation (P_{fix}), we apply the classical expression for populations with constant size [5], originally derived for Wright-Fisher and Moran models.

$$P_{fix} = \frac{1-(1+s)^{-1}}{1-(1+s)^{-N}} \tag{7}$$

  We further simplified this expression by taking the limit of large population size ($N \to \infty$) in the case of drivers and quasi-neutrality ($s_p \to 0$) in the case of passengers [2].

$$P_{fix} \approx \begin{cases} \frac{s_d}{1+s_d} & \text{for regular drivers} \\ \frac{\epsilon s_d}{1+\epsilon s_d} & \text{for enhanced drivers} \\ \frac{1}{N} + \mathcal{O}(s_p) & \text{for passengers} \end{cases} \tag{8}$$

  With these approximations, the rate of mutation accumulation becomes

$$\begin{array}{lll} v_d & = \mu(1-f_e)T_dN\frac{s_d}{1+s_d} & \text{for regular drivers} \\ v_\epsilon & = \mu f_e T_d N \frac{\epsilon s_d}{1+\epsilon s_d} & \text{for enhanced drivers} \\ v_p & = \mu T_p + \mathcal{O}(s_p) & \text{for passengers} \end{array} \tag{9}$$

- The different timescales of the population dynamics in the absence of new mutations (eqn. 6) and the mutation accumulation process (eqn. 9) allow us to approximate the dynamics of the cell population size in the presence of mutations as

$$\dot{N} = v_d \Delta N_d + v_\epsilon \Delta N_\epsilon + v_p \Delta N_p \tag{10}$$

  In this expression, ΔN_d, ΔN_ϵ, and ΔN_p represent the change in the equilibrium population size as a consequence of the fixation of a new regular driver, enhanced driver, and passenger, respectively. By combining eqns. 3 and 6 and comparing the equlibrium population sizes with n_d and n_d+1 regular drivers (same for enhanced drivers and passengers), we obtain that

$$\begin{array}{lll} \Delta N_d & = s_d N & \text{for regular drivers} \\ \Delta N_\epsilon & = \epsilon s_d N & \text{for enhanced drivers} \\ \Delta N_p & = -s_p N & \text{for passengers} \end{array} \tag{11}$$

1.2 Critical population size

In a previous work, McFarland et al. showed that, in the absence of epistasis, this model is characterized by the existence of a critical population size, N_c. If that critical population size is not reached, the tumor recedes with very high probability [1]. This phenomenon results from a tug-of-war between beneficial drivers and slightly deleterious passengers (this interpretation becomes clear from eqn. 10). If the population is sufficiently small, the rate at which new drivers appear and reach fixation is not enough to

compensate for the fitness loss due to the accumulation of passengers, which leads to the decay of the population. Here we use the same approximations as in [1] to obtain the critical population size in the presence of positive epistasis and show that its value is smaller than in the absence of epistasis. To obtain the critical population size, we replace eqns. 11 and 9 into eqn. 10. After grouping terms, we obtain:

$$\dot{N} = \mu T_p s_p N \left(\frac{N}{N_c'} - 1 \right) \tag{12}$$

where N_c' can be interpreted as a critical population size (see below) and has the following expression:

$$N_c' = \frac{N_c}{1 + f_e \left(\epsilon^2 \frac{1+s_d}{1+\epsilon s_d} - 1 \right)} \tag{13}$$

In the absence of epistasis ($f_e = 0$ or $\epsilon = 1$), the critical population size becomes

$$N_c = \frac{s_p T_p (1 + s_d)}{s_d^2 T_d} + \mathcal{O}(s_p^2) \tag{14}$$

As anticipated, $N_c' < N_c$ if $\epsilon > 1$ (positive epistasis). Notably, the solution of eqn. 12 is an increasing function of t (with $\lim_{t\to\infty} N(t) = \infty$) if $N(0) > N_c'$ and a decreasing function of t (with $\lim_{t\to\infty} N(t) = 0$) if $N(0) < N_c'$. Thus, in the deterministic version of the model described by eqn. 12, the critical population size separates tumors in two classes: those that grow indefinitely ($N > N_c'$) and those that become extinct ($N < N_c'$).

Although our notation does not make it explicit, it is important to note that, because in deriving eqn. 10 we assumed that the population size was always at equlibrium for a given mutational load, the variable N in eqns. 10-12 corresponds to the equilibrium states of eqn. 5 (denoted by N^* in eqn. 6). Therefore, the dynamics of the "population size" (N) in eqns. 10-12 represents, in fact, the temporal evolution of the effective carrying capacity of the system, that takes initial value N_0 (regardless of the actual population size at the beginning of the simulation) and changes with the mutational profile as BN_0. Consistent with this interpretation, stochasatic simulations starting from a single cell usually progress to cancer if the basal carrying capacity N_0 is greater than N_c' (except for a small probability of extinction of order $1/N$ in the few first generations) [2]. Similarly, simulations starting with a large number of cells usually become extinct if $N_0 < N_c'$. This happens because, as discussed above, the population typically reaches its equilibrium size $N^* = N_0$ before any drivers or passengers can reach fixation, effectively erasing any memory of the actual initial size.

1.3 Critical driver-to-passenger ratio

In this section, we derive a relationship for the ratio of drivers to passengers that separates growing from receding tumors. A similar expression was obtained by McFarland et al. [2] in the absence of epistasis. Here, we extend it to tumors with positive epistasis. We start from the expression for the equilibrium population size, $N^* = BN_0$ (eqn. 6):

$$N(n_d, n_\epsilon, n_p) = \frac{(1 + s_d)^{n_d} (1 + \epsilon s_d)^{n_\epsilon}}{(1 + s_p)^{n_p}} N_0 \tag{15}$$

Note that we have used N instead of N^* for simplicity and consistency with the notation used in eqns. 10-12. Now we impose the condition for tumor growth in the absence of epistasis, derived in the section 1.2:

$$\begin{aligned} N(n_d, n_\epsilon, n_p) &> N_c \qquad (16) \\ \log\left(N(n_d, n_\epsilon, n_p)\right) &> \log(N_c) \\ n_d \log(1 + s_d) - n_p \log(1 + s_p) + \log(N_0) &> \log(N_c) \\ n_d &> R_c(n_p) \end{aligned}$$

where

$$R_c(n_p) = \frac{\log(N_c/N_0)}{\log(1+s_d)} + \frac{\log(1+s_p)}{\log(1+s_d)}\, n_p \tag{17}$$

Eqn. 16-17 define a critical line, R_c, in the (n_p, n_d) plane, such that, in the absence of epistasis, mutation configurations above the line lead to tumor progression and those below the line do not [2].

We now intend to obtain an analogous expression that accounts for epistasis. To make it applicable to real-world scenarios in which the epistasis network may be unknown, the expression should include regular and enhanced drivers in a single variable, that we denote as $n_{d,\epsilon}$. To that end, we consider two extreme cases: (i) The probability of fixation of enhanced drivers is the same as regular drivers (this is true, for example, in the star before the trigger occurs). In that case, the fraction of enhanced and regular drivers is proportional to the fraction of cancer genes in and out of the epistasis network, that is $n_\epsilon = f_e n_{d,\epsilon}$ and $n_d = (1-f_e)n_{d,\epsilon}$. (ii) The probability of fixation of regular drivers is negligible with respect to enhanced drivers (this would occur, for example, in a strict two-hit scenario). In that case, $n_\epsilon = n_{d,\epsilon}$ and $n_d = 0$. The critical conditions derived from these two scenarios are, respectively,

$$n_{d,\epsilon} > \frac{\log(1+s_d)}{(1-f_e)\log(1+s_d) + f_e \log(1+\epsilon s_d)}\, R_c(n_p) \tag{18}$$

$$n_{d,\epsilon} > \frac{\log(1+s_d)}{\log(1+\epsilon s_d)}\, R_c(n_p) \tag{19}$$

In both scenarios, the factor to the left of R_c is smaller than 1. Therefore, epistasis reduces the critical number of drivers relative to passengers required for cancer progression by reducing the intercept and the slope.

1.4 Tumor rescue

Driver mutations in central genes of the epistasis network (trigger drivers) can facilitate tumor rescue by changing the critical population size from N_c to N'_c as the epistasis network "switches on". To illustrate that possibility, let us consider a population with basal carrying capacity $N_0 < N_c$. According to eqn. 12, the population will initially recede. Let us now suppose that a trigger driver reaches fixation at time t_D. From that moment on, drivers hitting any gene of the epistasis network will have an enhanced fitness effect ϵs_d and the dynamics of the population will be described by eqn. 12 with critical size N'_c (given by eqn. 13). Moreover, the fixation of the trigger driver produces a change ΔN_D in the population that we assume to be effectively instantaneous. If the new population size fulfils the condition $N(t_D)+\Delta N_D > N'_c$ the tumor will expand. We term this phenomenon tumor rescue.

Unlike other drivers and passengers, trigger drivers are unique random events whose study requires a probabilistic approach. Let $P(t_D = t)$ be the probability density function for the time at which the trigger driver reaches fixation. Following the previous argument, the probability of tumor rescue can be calculated as

$$P_{SOS} = \int_0^\infty P(t_D = t)\, H(N(t) + \Delta N_D - N'_c)\, dt \tag{20}$$

where $H(N(t_D)+\Delta N_D - N'_c)$ is the Heaviside step function and represents the condition for supercritical proliferation after the trigger mutation. To obtain the overall rescue probability, we need to calculate the tumor size just before the trigger driver, $N(t_D)$, the increase in tumor size due to the fixation of the trigger driver, ΔN_D and the probability that the trigger driver appears at any given time, $P(t_D = t)$. In the following subsections we will derive approximate expressions for these values.

1.4.1 Population size immediately before triggering

To calculate the population size at the trigger time, we reproduce the same line of reasoning that led us to derive eqns. 12-14 but with a subtle modification. Because we are conditioning the dynamics to not having a trigger, the target size for drivers must be modified to exclude every mutation that could act as a network-level trigger. In the case of cliques, that implies excluding driver mutations in any gene of the network, whereas in the case of stars, that implies excluding drivers in the hub. As a result, the pre-trigger target sizes for drivers in cliques and stars become, respectively

$$^{c}T_d = (1 - f_e)T_d \tag{21}$$

$$^{s}T_d = \left(1 - \frac{1}{G}\right) T_d \tag{22}$$

With these definitions, the dynamics of the population conditioned to the absemce of a trigger obeys the following differential equation

$$\dot{N} = \mu T_p s_p N \left(\frac{N}{^{x}N_c} - 1\right) \tag{23}$$

with $x \in \{c, s\}$ for cliques and stars, respectively, and

$$^{c}N_c = \frac{s_p T_p (1 + s_d)}{s_d^2 \, ^{c}T_d} + \mathcal{O}(s_p^2) \tag{24}$$

$$^{s}N_c = \frac{s_p T_p (1 + s_d)}{s_d^2 \, ^{s}T_d} + \mathcal{O}(s_p^2) \tag{25}$$

Integrating eqn. 23 from $t = 0$ to $t = t_D$ we obtain the expression for the population size at the trigger time:

$$^{x}N(t_D) = \frac{^{x}N_c}{1 + (^{x}N_c/N_0 - 1)e^{\mu T_p s_p t_D}} \tag{26}$$

1.4.2 Population size immediately after triggering

The next step is to calculate the population size immediately after the fixation of the trigger driver. We denote the post-trigger population size as $^{x}N_D$ and it is equal to the pre-trigger population size plus the increase in population size due to the trigger:

$$^{x}N_D = {^{x}N(t_D)} + {^{x}\Delta N_D} \tag{27}$$

with $x \in \{c, s\}$ for cliques and stars, respectively. The expression for cliques can be readily obtained by applying eqn. 11. In a clique, the trigger driver is the first driver hitting any gene from the network. In consequence, the immediate fitness effect of the trigger is just s_d (it is not enhanced by epistasis because there are no mutations in neighbor genes). For that reason, we have that

$$^{c}N_D = {^{c}N(t_D)} + {^{c}\Delta N_D} = {^{c}N(t_D)} + s_d \, {^{c}N(t_D)} = (1 + s_d) \, {^{c}N(t_D)} \tag{28}$$

The case of stars is more complicated. Indeed, by the time that a trigger driver hits the hub, other drivers can already be present in peripheral cancer genes. As a result, the fitness increase associated with the appearance of the trigger driver depends on the number of peripheral drivers n_ϵ. Taking that into account, the population size immediately after the trigger driver reaches fixation in stars networks becomes:

$$^{s}N_D = \begin{cases} (1 + s_d) \, {^{s}N(t_D)} & \text{if } n_\epsilon(t_D) = 0 \\ (1 + \epsilon s_d) \left(\frac{1+\epsilon s_d}{1+s_d}\right)^{n_\epsilon(t_D)} {^{s}N(t_D)} & \text{if } n_\epsilon(t_D) > 0 \end{cases} \tag{29}$$

where $n_\epsilon(t_D)$ is the number of peripheral drivers that have reached fixation at time t_D. (The first factor of the second case corresponds to the fitness effect of the trigger driver and the second factor accounts for the enhanced fitness effect of preexisting peripheral drivers.)

A rigorous study of the star would require reformulating eqn. 20 to consider cases with and without preexisting peripheral drivers and their respective probabilities. Instead, for simplicity, we replaced the random variable ${}^sN_D(={}^sN(t_D)+{}^s\Delta N_D)$ in the Heaviside step function of eqn. 20 by a weighted average $\langle {}^sN_D\rangle$ that takes into account the probabilities of having zero or more prefixated periphearal drivers. Although the error introduced by this approximation is difficult to estimate *a priori*, we validated this approach by comparing the resulting values of the rescue probability with those obtained thorugh simulations (S1 Fig, right). We calculated the weighted average of ${}^sN_D$ as

$$\langle {}^sN_D\rangle = \sum_{n_\epsilon=0}^{\infty} {}^sN_D(n_\epsilon)P(n_\epsilon) \tag{30}$$

where $P(n_\epsilon)$ is the probability to find n_ϵ prefixated drivers in peripheral cancer genes.

Because occurrence and fixation of peripheral drivers before triggering is independent of the presence of other peripheral drivers, $P(n_\epsilon)$ can be approximated by a Poisson distribution with mean $\lambda(t_D) = \mu f_e T_d \frac{s_d}{1+s_d}\int_{\tau=0}^{t_D} N(\tau)$. (Note that the expression of λ results from summing up the mean number of drivers per generation per cell for all cells in all generations, from $t=0$ until t_D.) As a result, the population size after fixation of the trigger driver, $\langle {}^sN_D\rangle$ becomes

$$\langle {}^sN_D\rangle = \left[(1+s_d)e^{-\lambda} + \sum_{n_\epsilon=1}^{\infty}(1+\epsilon s_d)\left(\frac{1+\epsilon s_d}{1+s_d}\right)^{n_\epsilon}\frac{e^{-\lambda}\lambda^{n_\epsilon}}{n_\epsilon!}\right]{}^sN(t_D) \tag{31}$$

(where we have omitted the explicit dependency of λ on t_D to lighten notation.) By defining $A=\frac{1+\epsilon s_d}{1+s_d}$ and operating:

$$\langle {}^sN_D\rangle = \left[(1+s_d)e^{-\lambda} + (1+\epsilon s_d)e^{(A-1)\lambda}\sum_{n_\epsilon=1}^{\infty}\frac{e^{-A\lambda}(A\lambda)^{n_\epsilon}}{n_\epsilon!}\right]{}^sN(t_D) \tag{32}$$

This expression can be simplified by taking into account that $\frac{e^{-A\lambda}(A\lambda)^{n_\epsilon}}{n_\epsilon!}$ corresponds to the probability mass function of a Poisson distribution with parameter $A\lambda$. As a result,

$$\langle {}^sN_D\rangle = \left[(1+s_d)e^{-\lambda} + (1+\epsilon s_d)e^{(A-1)\lambda}\left(1-e^{-A\lambda}\right)\right]{}^sN(t_D) \tag{33}$$

Finally, after some manipulation and replacing back A:

$$\langle {}^sN_D\rangle = \left[(1+\epsilon s_d)\,e^{(\epsilon-1)\,\frac{s_d}{1+s_d}\,\lambda} - (\epsilon-1)\,s_d\,e^{-\lambda}\right]{}^sN(t_D) \tag{34}$$

1.4.3 Critical trigger time

Because ${}^cN(t_D)$ is a decreasing function of t_D (eqn. 26), the post-trigger population size in the clique (${}^cN_D$ in eqn. 28) also decreases with t_D, tending to 0 as $t_D\to\infty$. As a result, the argument of the Heaviside function in eqn. 20 only changes its sign at most once, from being positive to being negative. The value of t_D at which the argument of the Heaviside step function becomes negative can be obtained by solving

$${}^cN_D(t_D) = N_c' \tag{35}$$

Let us define the critical trigger time t_c as the value of t_D that solves eqn. 35. Note that, if $t_c < 0$, the tumor cannot be rescued because ${}^cN_D < N'_c$ for any positive trigger time. If $t_c > 0$, we have that

$$H\big(N(t_D) + \Delta_D - N'_c\big) = \begin{cases} 1 & \text{if } t_D \leq t_c \\ 0 & \text{if } t_D > t_c \end{cases} \tag{36}$$

Therefore, the integral in eqn. 20 can be rewritten as

$$P_{SOS} = \int_0^{t_c} P(t_D = t)\, dt = P(t_D < t_c) \tag{37}$$

This formulation of the rescue probability highlights the fact that, in the deterministic approximation, rescue is only possible if the trigger driver occurs before t_c. This observation justifies the interpretation of t_c as a critical time.

Extnding these arguments to the star is not straightforward because the complex dependency between $\langle {}^sN_D \rangle$ and t_D (ultimately due to the accumulation of peripheral drivers over time) does not allow rule out the possibility that the argument of the Heaviside step function becomes negative for some $t_D < t_{min}$. However, we have numerically verified that in all the scenarios explored in this work, the equation ${}^sN_D(t_D) = N'_c$ has a single solution, with the argument of the Heaviside step function changing from positive to negative as t_D increases. In consequence, eqn. 37 also applies in the case of the star. To apply eqn. 37 to the star, t_c must be first obtained by numerically solving the folowing equation for the implicit variable t_D:

$$\langle {}^sN_D \rangle = N'_c \tag{38}$$

1.4.4 Rescue probability

It follows from eqn. 37 that the overall rescue probability P_{SOS} is equal to the probability that the trigger driver reaches fixation before the critical time t_c. By analogy with the arguments that led to eqn. 9, the rate at which trigger drivers appear and reach fixation is $v_D = \mu T_D \frac{s_D}{1+s_D} N(t)$, where T_D is the mutational target size associated with the trigger driver ($f_e T_d$ in cliques and T_d/G in stars) and s_D is the fitness effect of the trigger driver (whose possible values are derived below). If we consider trigger fixation as a stochastic process with rate q, the probablity that no driver has reached fixation after a time t obeys the following differential equation

$$\frac{dP_{no\_trigger}(t)}{dt} = -q(t) P_{no\_trigger} \tag{39}$$

(note the dependency of $q(t)$ with t through $N(t)$ and, in the case of the star, s_D, as described below.) By integrating this equation, we obtain a general expression for the probability that the trigger driver occurs before a given time t.

$$P(t_D > t) = \exp\left\{ - \int_0^t q(u) du \right\} \tag{40}$$

$$P(t_D \leq t) = 1 - \exp\left\{ - \int_0^t q(u) du \right\} \tag{41}$$

$$\tag{42}$$

Replacing t by t_c and the full expression for q we get

$$P_{SOS} = P(t_D \leq t_c) = 1 - \exp\left\{ -\mu T_D \int_{\tau=0}^{t_c} N(\tau) \frac{s_D}{1+s_D} \right\} \tag{43}$$

The fitness effect s_D is, by definition, the solution of the equation

$$(1 + s_D)B = B' \tag{44}$$

where B and B' are the birth rates before and after acquiring the trigger driver, respectively. According to eqn. 3, the fitness effect of the trigger driver in clique (${}^c s_D$) and star (${}^s s_D$) epistasis networks are

$$ {}^c s_D = s_d \tag{45}$$

$$ {}^s s_D = \begin{cases} s_d & \text{if } n_\epsilon = 0 \\ (1+\epsilon s_d)\left(\frac{1+\epsilon s_d}{1+s_d}\right)^{n_\epsilon} - 1 & \text{if } n_\epsilon > 0 \end{cases} \tag{46}$$

As previously discussed in the context of eqn. 29, ${}^s s_D$ depends on the presence of drivers in peripheral genes of the star when the trigger occurs. Moreover, ${}^s s_D$ implicitly varies with time because the probability to have n_ϵ periphearal drivers, $P(n_\epsilon)$ varies with time. As we did for ${}^s N_D$ (eqn. 30), to calculate P_{SOS} in the star we replaced the term $\frac{s_D}{1+s_D}$ by a weighted average $\langle \frac{s_D}{1+s_D} \rangle$ that takes into account the probability to find preexistent peripheral drivers. The error introduced by this approximation is difficult to evaluate *a priori*, although a comparison of the final estimates of P_{SOS} and the values obtained from simulation indicates that, within the range of parameters explored in this study, the differences are generally small (S1 Fig, right). We calculated the weighted average of $\left\langle \frac{{}^s s_D}{1+{}^s s_D} \right\rangle$ as

$$\left\langle \frac{{}^s s_D}{1 +^s s_D} \right\rangle = \sum_{n_\epsilon=0}^{\infty} \frac{{}^s s_D}{1 +^s s_D} P(n_\epsilon) \tag{47}$$

$$\begin{aligned} \left\langle \frac{{}^s s_D}{1 +^s s_D} \right\rangle (t) = 1 &- \frac{(\epsilon - 1)s_d}{(1+s_d)(1+\epsilon s_d)} \exp\left\{ -\mu T_d f_e \frac{s_d}{1+s_d} \sum_{k=0}^{t-1} N(k) \right\} \\ &- \frac{1}{1+\epsilon s_d} \exp\left\{ -\frac{(\epsilon-1)s_d}{(1+s_d)(1+\epsilon s_d)} \mu T_d f_e \frac{s_d}{1+s_d} \sum_{k=0}^{t-1} N(k) \right\} \end{aligned} \tag{48}$$

where, as in eqn. 31-34, we assumed that $P(n_\epsilon)$ follows a Poisson distribution with mean $\lambda(t_D) = \mu f_e T_d \frac{s_d}{1+s_d} \int_{\tau=0}^{t_D} N(\tau)$.

1.5 Critical rescue time for population doubling

The rescue of the tumor has been defined in terms of reaching a size greater than the post-trigger critical size N'_c. However, it is possible that, even fulfilling this condition, the growth rate of the rescued tumor is too slow to be clinically relevant. To account for that possibility, in the simulations we only classified the outcome as tumor progression if the size doubled before $t_{max} = 30000$ generations. From an analytical perspective, this condition sets a second critical time t'_c. Rescue must occur before t'_c so that there is enough time for the population to double its size before reaching t_{max}. The value of t'_c is obtained by applying eqn. 26 (that describes the change in population size with time) with the population at the critical rescue time as initial condition, making it equal to $2N_0$, and numerically solving the resulting equation:

$$\frac{N'_c}{1 + \left(\frac{N'_c}{\langle {}^x N_D(t'_c) \rangle} - 1\right) \exp\{\mu T_p s_p (t_{max} - t'_c)\}} = 2N_0 \tag{49}$$

Finally, the probability that the population is rescued and doubles its size before t_{max} is calculated by applying eqns. 43, 45 and 48 to the smallest of t_c and t'_c.

References

1. McFarland, C.D., Korolev, K.S., Kryulov, G.V., Sunyaev, S.R. & Mirny, L.A. Impact of deleterious passenger mutations on cancer progression. *Proc. Natl. Acad. Sci. USA*, **110**, 2910-5 (2013).

2. McFarland, C.D., Mirny, L.A. & Korolev, K.S. Tug-of-war between driver and passenger mutations in cancer and other adaptive processes. *Proc. Natl. Acad. Sci. USA*, **111**, 15138-43 (2014).

3. Tomasetti, C. & Vogelstein, B. Cancer etiology. Variation in cancer risk among tissues can be explained by the number of stem cell divisions. *Science*, **347**, 78-81 (2015).

4. Gao, Z., Wyman, M.J., Sella, G. & Przeworski, M. Interpreting the Dependence of Mutation Rates on Age and Time. *PLoS Biol*, **14**, e1002355 (2016).

5. Nowak, M.A. *Evolutionary Dynamics: Exploring the Equations of Life*, Harvard University Press (2006).
