# Supplementary figures and images for: Synergistic epistasis among cancer drivers can rescue early tumors from the accumulation of deleterious passengers
Source: PLoS Comput Biol. 2024 Apr 30;20(4):e1012081. doi: 10.1371/journal.pcbi.1012081 (PMC11087069; doi:10.1371/journal.pcbi.1012081)

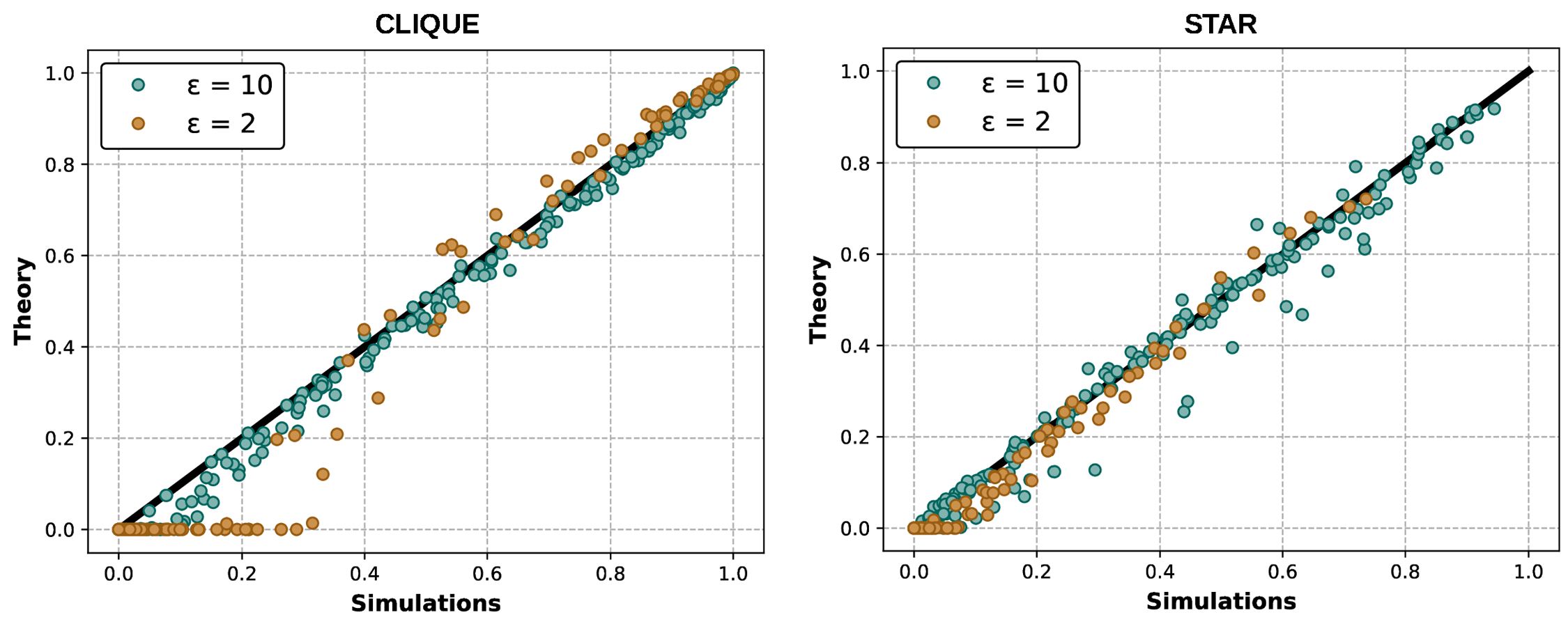

Supplement: S1 Fig — The plot compares the rescue probabilities obtained for all parameter values explored in Figs 4 and S2. The overall coefficient of determination, calculated by taking the values from simulations as the “true” ones, is R2 = 0.987 (n = 1056). The coefficients of determination for each dataset are: clique with ϵ = 2, R2 = 0.962; clique with ϵ = 10, R2 = 0.994; star with ϵ = 2, R2 = 0.978; star with ϵ = 10, R2 = 0.984. The lower accuracy of the analytical approximation in cliques with ϵ = 2 results from the occasional rescue of tumors after the critical time (see section 1.4.3 in S1 Text). Such “late” rescue can occur if stochastic fluctuations in the rate at which drivers accumulate lead to fixation of an enhanced driver shortly after the trigger. These stochastic effects are more relevant in conditions in which the trigger driver only produces a modest increase in the population size (ΔND, see section 1.4.2 in S1 Text) and the critical population size after rescue, Nc′, is relatively close to Nc. Both conditions are especially met by cliques (due to the absence of preexistent drivers in the epistasis network, that leads to relatively small ΔND) with low epistasis factors (that lead to modest decreases in Nc′). Even in those cases, despite the systematic bias in analytical approximations, the analytical expression can distinguish between scenarios of high and low rescue probabilities (only 1.3% of the data points have rescue probability >0.5 in the simulations and <0.5 in the analytical expression or vice versa). (TIF) [file pcbi.1012081.s002.tif]

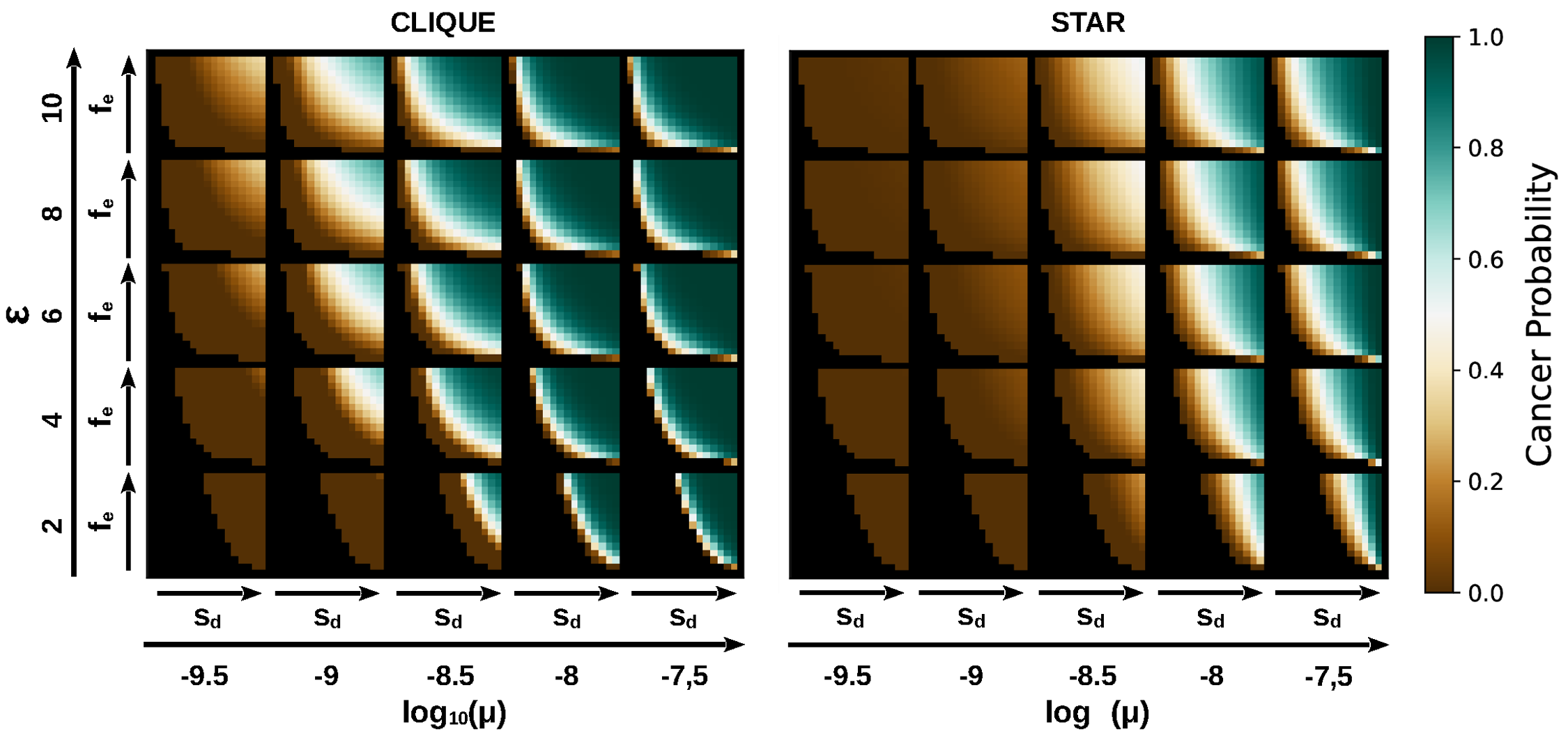

Supplement: S2 Fig — (A) Analytical probability of tumor progression as a function of network structure, mutation rate (μ), driver fitness effect (sd), fraction of genes subject to epistasis (fe), and strength of epistasis (ϵ). (TIF) [file pcbi.1012081.s003.tif]

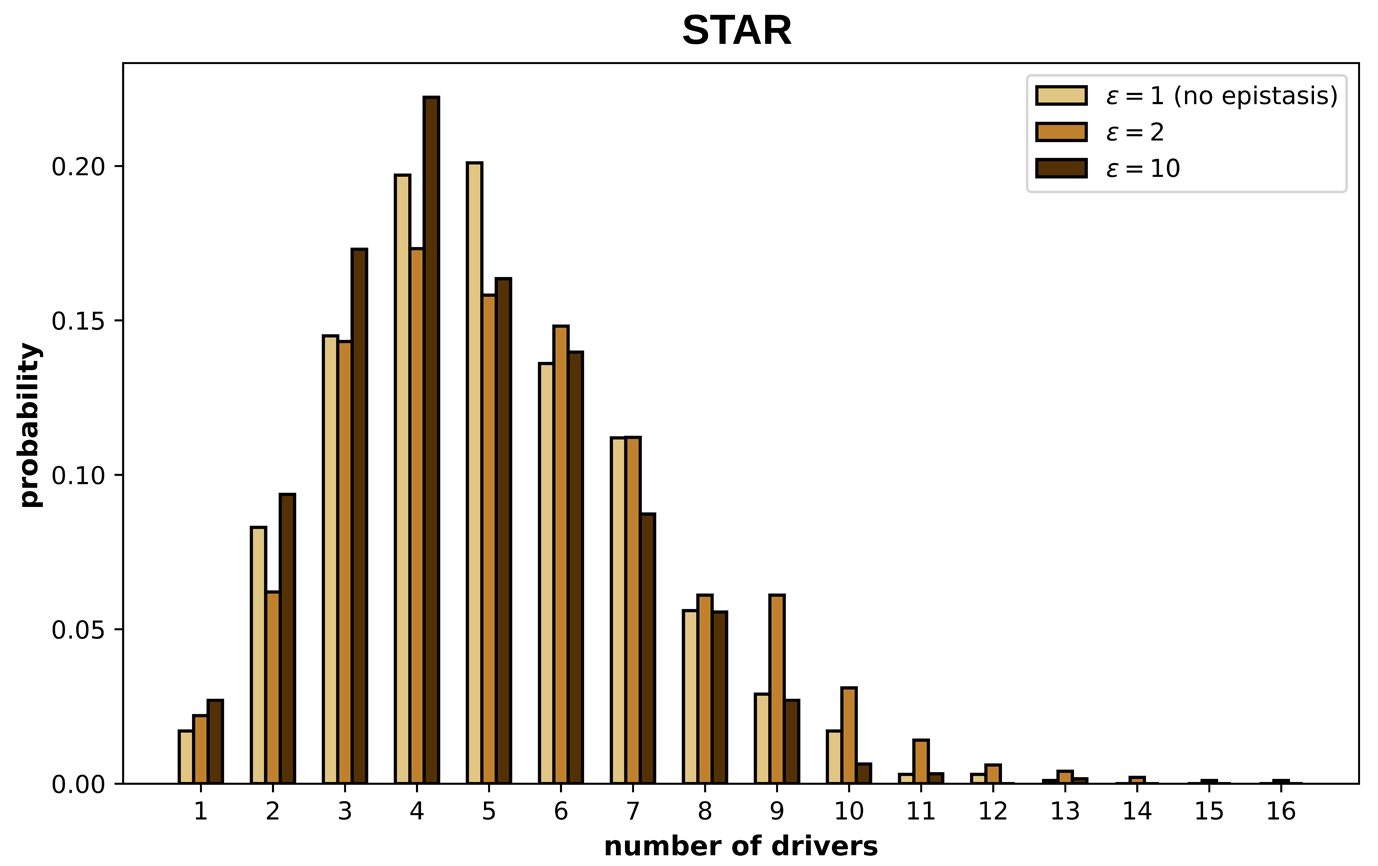

Supplement: S3 Fig — The distributions correspond to the maximum number of drivers observed along 1000 trajectories that did not double their initial size in 30000 cell divisions, considering a star-like epistasis network (see Fig 7 for a clique-like network). Parameter values: μ = 5×10^−9^, sd = 0.05, fe = 0.5, ϵ = 2, rest of parameters as in Fig 3. (TIF) [file pcbi.1012081.s004.tif]
